# CerevianNet: parameter efficient multi-class brain tumor classification using custom lightweight CNN

**DOI:** 10.3389/fmed.2025.1664673

**Published:** 2026-01-23

**Authors:** Md Khurshid Jahan, Abdullah Al Shafi, Maher Ali Rusho, Md. Shahriar Hussain, Ahmed Faizul Haque Dhrubo

**Affiliations:** 1 Department of Electrical and Computer Engineering, North South University, Dhaka, Bangladesh; 2 Department of Lockheed Martin Engineering Management, University of Colorado Boulder, Boulder, CO, United States

**Keywords:** brain tumor, custom lightweight CNN, MRI, medical imaging, light weight

## Abstract

Brain tumors are a life-threatening condition, and their early detection is crucial for effective treatment and improved survival rates. Traditional manual evaluation techniques, such as expert radiologist assessments and visual inspections, are widely used for diagnosing brain tumors. While these methods can be highly reliable, they are often time-consuming, prone to human error, and challenging to scale for large datasets. Consequently, there is a growing demand for Computer-Aided Diagnostic (CAD) systems to overcome these limitations and deliver fast, accurate, and scalable solutions. Despite these promising advancements, the study highlights potential limitations, including susceptibility to overfitting due to the limited availability of labeled data and the need for extensive hyperparameter tuning to generalize across diverse datasets. This study proposes a scalable multi-class brain tumor classification framework optimized for small-form-factor devices. We introduced a novel, lightweight custom convolutional neural network (CNN) that maintains high classification accuracy while significantly reducing computational complexity. We evaluated the model's capacity by training and testing it on five different datasets, and it performed well on all five. We observed a significant improvement in performance with the model on larger datasets, but it struggled with smaller and imbalanced datasets. We achieved significant scores on the datasets, and we had the highest testing accuracy on Dataset-5 (99.67% training accuracy, 98.17% validation accuracy, and 98.30% testing accuracy). What is important to note is that we had the lowest testing accuracy on Dataset-3 (99.99% training accuracy, 74.11% validation accuracy, and 75.63% testing accuracy). The proposed framework leverages state-of-the-art pretrained deep learning models, including EfficientNetb3, ResNet-101, ResNet-50, Xception, AlexNet, DenseNet121, Swin Transformer, and our custom lightweight CNN model. Experimental evaluations demonstrate that EfficientNetb3 achieves the highest accuracy of 99.11%, while the custom lightweight CNN attains 98% accuracy with 4.1 × fewer parameters and reduced training time. These results highlight the effectiveness of computer-aided approaches in achieving near-expert performance, making them suitable for integration into clinical workflows. This research paves the way for deploying efficient and scalable deep learning models in real-world medical applications, thereby expanding accessibility to accurate brain tumor diagnosis.

## 1 Introduction

Brain tumors represent a critical medical challenge, with an estimated 300,000 new cases identified globally each year (1). These tumors, which can be classified as either benign or malignant, cause significant health concerns due to their complex nature and potential to impact various brain functions. Malignant tumors are especially worrisome because they tend to grow aggressively and can metastasize to other regions of the brain and body, unlike benign tumors that typically remain localized. Imaging technologies such as magnetic resonance imaging (MRI) and computed tomography (CT) scans play a pivotal role in diagnosing and assessing these tumors, allowing for early detection and follow-up, which are essential for effective treatment (2). The brain is a vital organ of the central nervous system (CNS),and it is responsible for controlling physical, cognitive, and emotional functions; any anomaly in its structure can have profound implications. The CNS comprises intricate networks of neurons that connect the brain and spinal cord, forming a continuous communication pathway. Detecting and diagnosing brain tumors early is crucial, as these abnormalities can disrupt these networks, significantly impacting bodily functions. Given the high-stakes nature of brain tumor diagnosis, medical imaging modalities like MRI are preferred for their precision and safety, as MRI does not involve harmful radiation (3). MRI images, including FLAIR, T1, T1 contrast-enhanced, and T2-weighted images, enable detailed visualization of brain tissues, supporting radiologists in identifying tumor types based on their unique characteristics, such as shape, size, and location (4).

The Input Enhanced Vision Transformer (IEViT) expands the Vision Transformer (ViT) architecture by using a Convolutional Neural Network (CNN) block. This CNN block processes the input image in full, generating an image embedding that is added back into each transformer encoder layer iteratively. The patches of image are embedded and fed into a sequence of transformer layers, with the CNN embedding maintaining the image context. The inclusion of the CNN embedding improves the model, especially in classifying chest X-ray images, by providing a stacked feature representation that allows greater generalization beyond the training set (5). The DeepMedic neural network is a model based on three-dimensional (3D) CNNs architecture aimed for efficient and precise brain lesion segmentation (6). The DeepMedic model features a multi-scale approach using two parallel paths of convolutions that process all input masks concurrently before combining the feature maps in the segmentation step. The volume of information altered by multiple convolutions can also be reduced—thanks to the residual connections, which help maintain some of the information flow, as convolution is a lossy compression of the features. The additional features to the DeepMedic model are what allow DeepMedic to learn and compute segmentation maps efficiently.

The TransBTS model for 3D brain tumor segmentation integrates transformer and 3D CNN within an encoder–decoder framework. The encoder utilizes 3D convolutions to extract volumetric features, followed by reshaping these features and feeding them into a transformer for global context modeling. The decoder progressively upsamples these features to generate high-resolution segmentation maps. This hybrid architecture effectively captures both local and global dependencies in MRI brain tumor segmentation, improving performance compared to traditional CNN methods (7). The U-Transformer (8) model enhances the traditional UNet architecture by integrating transformer-based attention mechanisms for medical image segmentation. It uses two key attention modules: self-attention (Multi-Head Cross-Attention [MHSA]) in the encoder to capture global context and long-range dependencies across the image, and cross-attention (Multi-Head Cross-Attention [MHCA]) in the decoder to refine high-resolution features from the skip connections. These attention mechanisms help improve segmentation performance by addressing the limitations of local context in UNet, particularly for complex anatomical structures with low contrast. The model demonstrates significant performance improvements on abdominal CT images compared to the standard UNet.

The Multimodal Encoding-Decoding Network with Transformer (MEDT) model for multimodal sentiment analysis integrates natural language, visual, and acoustic data to address the challenges of long-term dependencies and dynamic interactions between modalities. The model consists of two main parts: a unimodal encoder that handles each modality separately using Bidirectional Encoder Representations from Transformers (BERT) for text and a transformer encoder for visual and acoustic data, and a multimodal joint-decoder that fuses the modality-specific features using a cross-modal attention mechanism (9). The Swin Transformer is available in several variants, each with different model sizes and layer configurations. The Swin-T (Tiny) variant has 29 M parameters, with layer numbers of 2, 2, 6, and 2 across four stages and a hidden dimension (C) of 96. The Swin-S (Small) variant increases the number of parameters to 50 million, with two layers each in stages 1 and 2, 18 layers in stage 3, and two layers in stage 4, while maintaining a hidden dimension of 96. The Swin-B (Base) model, designed for more demanding tasks, has 88 M parameters, with the same layer configuration as Swin-S, but a larger hidden dimension of 128. Finally, the Swin-L (Large) variant boasts 197 M parameters, with the same layer structure as the previous models but an even larger hidden dimension of 192. These configurations strike a balance between model performance and computational efficiency, making the Swin Transformer suitable for a wide range of vision tasks (10). The Refined UNet Lite (11) model is a lightweight and efficient architecture designed for edge-precise cloud detection in remote sensing images. It consists of a UNet backbone that includes four down-sampling blocks [Convolution-rectified linear unit (ReLU)-MaxPooling] for feature extraction, followed by four up-sampling blocks (UpSampling-Convolution-ReLU) to restore the spatial resolution of the feature maps. This backbone produces a coarse segmentation map of cloud regions. Afterward, a guided-filter layer is applied to refine the edges of the segmented cloud regions, improving the precision of the edge boundaries. The model is fully differentiable, enabling end-to-end training, and its lightweight design ensures faster processing times with high precision, particularly for detecting cloud edges in large-scale remote sensing datasets. The architecture is designed for efficiency, significantly reducing computational time while achieving effective edge-precise cloud detection.

The FD-MobileNet (Fast-Downsampling MobileNet) model introduces an efficient and optimized architecture designed for very limited computational resources (10–140 mega floating-point operations per second [MFLOPS]). The model employs a fast downsampling strategy, reducing computational cost by performing 32 × downsampling within the first 12 layers, which is only half the number of layers compared to the original MobileNet. This strategy significantly reduces the spatial dimensions early on, allowing more channels to be used in the later layers, thus increasing the information capacity and enhancing performance. The architecture includes 24 layers in total, comprising standard convolutions, depthwise separable convolutions, and fully connected layers. The model also employs depthwise separable convolutions, which reduce the number of parameters and computation time while maintaining performance. In terms of optimizations, FD-MobileNet achieves better inference speed compared to other networks like ShuffleNet, demonstrating a 1.11 × speedup over MobileNet and 1.82 × over ShuffleNet under the same computational budget (12). The GhostNet (13) architecture uses a model called the Ghost module, which produces greater feature maps using fewer parameters through intrinsic feature maps that perform the same linear operation as ordinary convolutions. The Ghost module decomposes the original convolution into two sections: an ordinary convolution, which produces a small even number of intrinsic feature maps with the same convoluted kernel, and a collection of linear operations that produce an arbitrarily larger number of “ghost” feature maps. Compared to a single ordinary convolution, GhostNet's Ghost module significantly reduces the number of parameters and floating-point operations per second (FLOPS). GhostNet consists of Ghost bottlenecks, which stack these Ghost modules, and is similarly structured to MobileNetV3, using Ghost bottlenecks instead of the original bottleneck blocks. This experimentation allows us to achieve dense computational cost while maintaining or increasing recognition performance, where GhostNet achieves 75.7% top-1 accuracy on ImageNet with 226 MFLOPS. We can also use width multipliers to further explore potential model configurations that adjust the model's size while balancing computational cost and accuracy. This demonstrates that our model outperforms MobileNetV3 and all other compact architectures on mobile devices, providing a fulfilling compromise with a relative speed/accuracy trade-off.

The SqueezeNet architecture is designed to maintain AlexNet-level accuracy with significantly fewer parameters. The core innovation of SqueezeNet is the Fire module, which consists of a squeeze layer (using 1 × 1 filters) followed by an expand layer that combines both 1 × 1 and 3 × 3 filters. This structure enables SqueezeNet to reduce the number of parameters by prioritizing 1x1 filters, which are computationally more efficient, while still capturing spatial information with the 3x3 filters. The model comprises 8 Fire modules and concludes with a global average pooling layer, followed by the final classification layer. The architecture achieves 57.5% top-1 accuracy on ImageNet with a 4.8 MB model size, a 50 × reduction in parameters compared to AlexNet. Further compression techniques, such as Deep Compression, reduce the model size to as low as 0.47 MB, maintaining similar accuracy (14). The EfficientNet (15) architecture is designed around a compound scaling method, which uniformly scales the network depth, width, and resolution with a fixed ratio to improve both accuracy and efficiency. The baseline model, EfficientNet-B0, is optimized through neural architecture search, using a Mobile Inverted Bottleneck (MBConv) and squeeze-and-excitation optimizations. It consists of multiple stages, starting with a 3 × 3 convolution and progressing through multiple MBConv layers with various kernel sizes (3 × 3, 5 × 5). The architecture is then scaled up from EfficientNet-B0 to B7 using the compound scaling method, achieving state-of-the-art accuracy (84.4% top-1) with 66 M parameters in EfficientNet-B7, significantly reducing the model size and FLOPS compared to previous architectures, such as GPipe and ResNet. The model's efficiency is evident as it is up to 8.4 × smaller and 6.1 × faster in inference than traditional models with similar accuracy, making it highly optimized for both accuracy and computational efficiency.

In recent years, Artificial Intelligence (AI) and Computer-Aided Diagnostic (CAD) systems have emerged as valuable tools for healthcare professionals, particularly in the automated detection and classification of brain tumors. Traditional machine learning techniques have been widely applied in CAD systems, relying on handcrafted features and classifiers like Support Vector Machines (SVM), Decision Trees (DT), and k-Nearest Neighbor (KNN) algorithms (16). However, feature engineering in these methods demands extensive domain knowledge and can be labor-intensive, especially with large datasets. With the advent of deep learning, they have gained popularity in medical imaging for their ability to automatically extract meaningful features from raw images, reducing the need for manual feature engineering (17). Despite the promise of CNNs, these models require substantial amounts of data to achieve optimal performance, presenting challenges in medical imaging where labeled datasets are limited. Moreover, the computational resources required for CNN training can be prohibitive. However, the benefits of automated feature extraction and the potential for accurate classification continue to drive the adoption of deep learning methods in brain tumor diagnosis.

The structure of this study is organized as follows. Section 2 reviews current state-of-the-art methodologies. Section 3 explains the methodology and workflow of the proposed model. Section 4 outlines the results achieved by the proposed model and compares them with those of the existing state-of-the-art approaches, while Section 5 outlines the future work of this study. Finally, Section 6 concludes the study by providing a rationale for the results.

## 2 Related work

Brain tumor classification is a critical aspect of diagnosing and treating brain tumors effectively. Various deep learning models have been proposed to accurately classify brain tumors using MRI images. Studies have shown the effectiveness of models like ResNet50V2, InceptionResNetV2, and DenseNet201 in achieving high accuracy rates ranging from 98.72 to 99.68% (18). Additionally, the use of pretrained models like VGG19 and ResNext101 32 × 8d has demonstrated testing accuracies of up to 100% in distinguishing between different types of brain tumors, including pituitary and glioma (19). Furthermore, the integration of computational intelligence and statistical image processing techniques has been instrumental in developing efficient frameworks for brain tumor segmentation and classification, with models like 3D-UNet and CNNs showcasing superior performance compared to existing techniques (20, 21). Zahoor introduced Res-BRNet, a novel deep residual and region-based CNN architecture for brain tumor classification using MRI scans. The model combines spatial and residual blocks to extract boundary-related features and texture variations. It was tested on datasets from Kaggle, Br35H, and Figshare. The dataset contains meningioma, glioma, pituitary tumors, and healthy images. Res-BRNet achieved exceptional performance with 98.22% accuracy, sensitivity of 0.9811, and F1-score of 0.9841. Their approach outperformed standard CNNs. The innovative architecture addresses the complexity of brain tumors, offering a promising tool to improve clinical diagnosis and treatment planning (22). Hong addressed challenges in brain tumor detection by proposing an improved Vision Transformer-based algorithm for classification. To enhance small datasets, techniques such as Homomorphic Filtering and Contrast Limited Adaptive Histogram Equalization (CLAHE) were applied. It improved data quality and model generalization. A novel relative position encoding method was introduced to enhance the Vision Transformer's self-attention mechanism, while residual structures in the Multi-Layer Perceptron ensured faster convergence and higher accuracy. Tested on an augmented open-source dataset, and the model achieved 91.36% accuracy. It surpassed VIT-B/16 by 5.54% (23). Sandhiya and her team proposed an enhanced brain tumor classification model using MRI images to assist medical professionals. Deep learning architectures, such as Inception V3 and DenseNet201, are utilized to extract features. These are combined with radiomic properties for improved accuracy. PSO-KELM classifies tumors into four categories: No Tumor, Gliomas, Meningiomas, and Pituitary Tumors. The model outperforms existing approaches on two benchmark datasets, achieving classification accuracies of 96.17 and 97.92% during training and 97.97 and 98.21% during testing. These results demonstrate significant advancements in brain tumor diagnosis and classification accuracy (24).

Various machine learning approaches have been employed to classify brain tumors, particularly high-grade malignant tumors, into specific classes with high precision, achieving mean average classification accuracies of 88.43% (KNN), 92.5% [multi-class Support Vector Machines (mSVM)], and 95.86% [Nearest Neighbor (NN)] (25). Raza et al. propose a hybrid deep learning model, DeepTumorNet, for classifying glioma, meningioma, and pituitary brain tumors using a modified GoogLeNet architecture. They replaced the last 5 layers with 15 new layers and used a leaky ReLU activation function to enhance the model's expressiveness. Tested on a public dataset, DeepTumorNet achieved 99.67% accuracy outperforming state-of-the-art models like AlexNet, ResNet50, and MobileNetv2 (26). Kang et al. propose a brain tumor classification method using an ensemble of deep features and machine learning classifiers. They employ transfer learning with several pretrained deep CNNs to extract features, and the top three are concatenated and fed into various classifiers. Testing on three open-access MRI datasets. The ensemble approach significantly improves performance with SVM with RBF kernel outperforming other classifiers (27). The authors present a comparative performance analysis of transfer learning-based VGG-16, ResNet-50, and Inception-v3 models. They demonstrate these pretrained models on a dataset of 233 MRI brain tumor images, aiming to locate tumors with the VGG-16 model. The performance is evaluated based on accuracy, indicating that the VGG-16 model achieves highly adequate accuracy rates in training and validation (28). The authors present an automated brain tumor detection method using the Unet architecture with ResNet50 as a backbone on the Figshare dataset. Multi-classification was performed with evolutionary algorithms and reinforcement learning through transfer learning. Other methods applied include ResNet50, DenseNet201, MobileNet V2, and InceptionV3, with NASNet achieving the highest accuracy at 99.6%, outperforming the state-of-the-art models (29). The main contribution of our study:

- Managing the challenge of resource-constrained devices by using a parameter-efficient, lightweight, and robust model.
- Proposed a custom CNN architecture optimized for faster inference and reduced computational complexity while maintaining high classification accuracy.
- Offering a comprehensive comparison of state-of-the-art pretrained models, highlighting the trade-offs between performance and computational efficiency.

Table 1 shows a comparison of different deep learning models in relation to the specifications, training environments, total parameters, and accuracy. The Pyramid Pooling Transformer (P2T) has a large architecture that uses 54.5 M parameters and 9.8 G FLOPS. When trained on ImageNet-1K, ADE20K, and MS-COCO, the top-1 accuracy is 87.9%. While the Pyramid Vision Transformer v2 (PVT v2) has 82.0 M parameters and 11.3 G FLOPS, it achieves an accuracy of 83.8% on the same datasets. The Swin-Unet is specifically for medical imaging segmentation, for example, Synapse Multi-Organ, Automated Cardiac Diagnosis Challenge with 4 M parameters with top-1 accuracy of 79.7% and EfficientFormer with 8.3 M parameters with an accuracy of 83.3%, when trained on datasets like ImageNet-1K, Ade20K, and MS-COCO. NASNet is designed for attention-based segmentation tasks, including rain streak removal and mist removal, and features 6.5 M parameters, achieving a Peak Signal-to-Noise Ratio (PSNR) of 43.50 and a Structural Similarity Index Measure (SSIM) of 0.9879. In contrast, for GhostNet, a monitoring model with 8 M parameters achieves an accuracy score of 76.3% when trained on indoor monitoring, outdoor monitoring, field monitoring for printed images, and drone images. The SA-Net, which uses Shuffle Attention for deep convolutional networks, also has 8 M parameters and achieves an accuracy of 76.3%, trained on datasets like ImageNet-1K and MS COCO. In medical image segmentation, the DS-TransUNet: Dual Swin Transformer UNet model performs well, achieving an F1 score of 0.92 and a mean Intersection over Union (mIoU) of 0.86 on polyp segmentation tasks. Finally, EfficientNetV2, a model optimized for training efficiency, uses 22 M parameters and 8.8B FLOPS, achieving 83.9% accuracy on datasets like ImageNet ILSVRC2012, CIFAR-10, CIFAR-100, Cars Dataset, and Flowers Dataset.

**Table 1:** Comparison of model performance, datasets, and parameters across various architectures.

**Model name**	**Dataset**	**Parameter**	**Accuracy**
Pyramid pooling transformer (P2T)	ImageNet-1K, ADE20K, MS-COCO	P2T-Large: 83.9%	P2T-large: parameters: 54.5 M and FLOPS: 9.8G
Pyramid Vision Transformer v2 (PVT v2)	ImageNet-1K, COCO 2017, ADE20k	PVT v2-B5 achieved 83.8%	PVT v2-B5: 82.0 M parameters, 11.8 GFLOPS
Swin-Unet	Synapse multi-organ segmentation dataset, automated cardiac diagnosis challenge dataset	Swin-Unet achieved 79.13%	Swin-Unet: 4 M parameters
EfficientFormer	ImageNet-1K, COCO 2017, ADE20k	EfficientFormer-L7 accuracy 83.3%	EfficientFormer-L7: 82.1 M parameters
Neuron attention stage-by-stage net (NASNet)	Rain100L, Rain100H, SPA-REAL, RainCityScapes, MPID	PSNR: 43.50 and SSIM: 0.9879	Parameters: 6,508,931
GhostNet	Indoor monitoring, outdoor monitoring, field monitoring, drone-captured images, network images	Achieves 97.45% mAP	Parameters: 2.75 M
SA-Net: shuffle attention for deep convolutional neural networks	ImageNet-1K, MS COCO	Accuracy: 76.38%	Parameters: 25.557 M
DS-TransUNet: dual swin transformer UNet	Polyp segmentation, ISIC 2018, GLAS, 2018 data science BOWL	Achieved an F1 score of 0.9219 and an mIoU of 0.8612	88 M parameters
EfficientNetV2	ImageNet ILSVRC2012, CIFAR-10, CIFAR-100, cars dataset, flowers dataset	Accuracy: 83.9%	Parameters: 22 M and FLOPS: 8.8B

## 3 Methodology

The proposed workflow adopts a systematic pipeline for medical image analysis. Started with preprocessing to standardize raw imaging data. Techniques such as noise reduction, spatial resizing, and intensity normalization are applied to minimize artifacts and enhance feature visibility. The curated dataset is then partitioned into training, validation, and test subsets using stratified splitting. This ensured balanced representation of tumor stages and sizes to mitigate bias. Several pretrained models were fine-tuned on the MRI dataset. Furthermore, A custom lightweight CNN architecture is subsequently trained on the preprocessed images, optimizing hyperparameters to maximize discriminative power across tumor classes. Model performance is rigorously evaluated using established metrics. Several metrics are used with emphasis on distinguishing subtle inter-class variations in tumor morphology. The optimal model is selected based on its generalizability to diverse tumor phenotypes and computational efficiency, prioritizing clinical applicability in diagnostic workflows.

### 3.1 Dataset description

**Figure 1:**
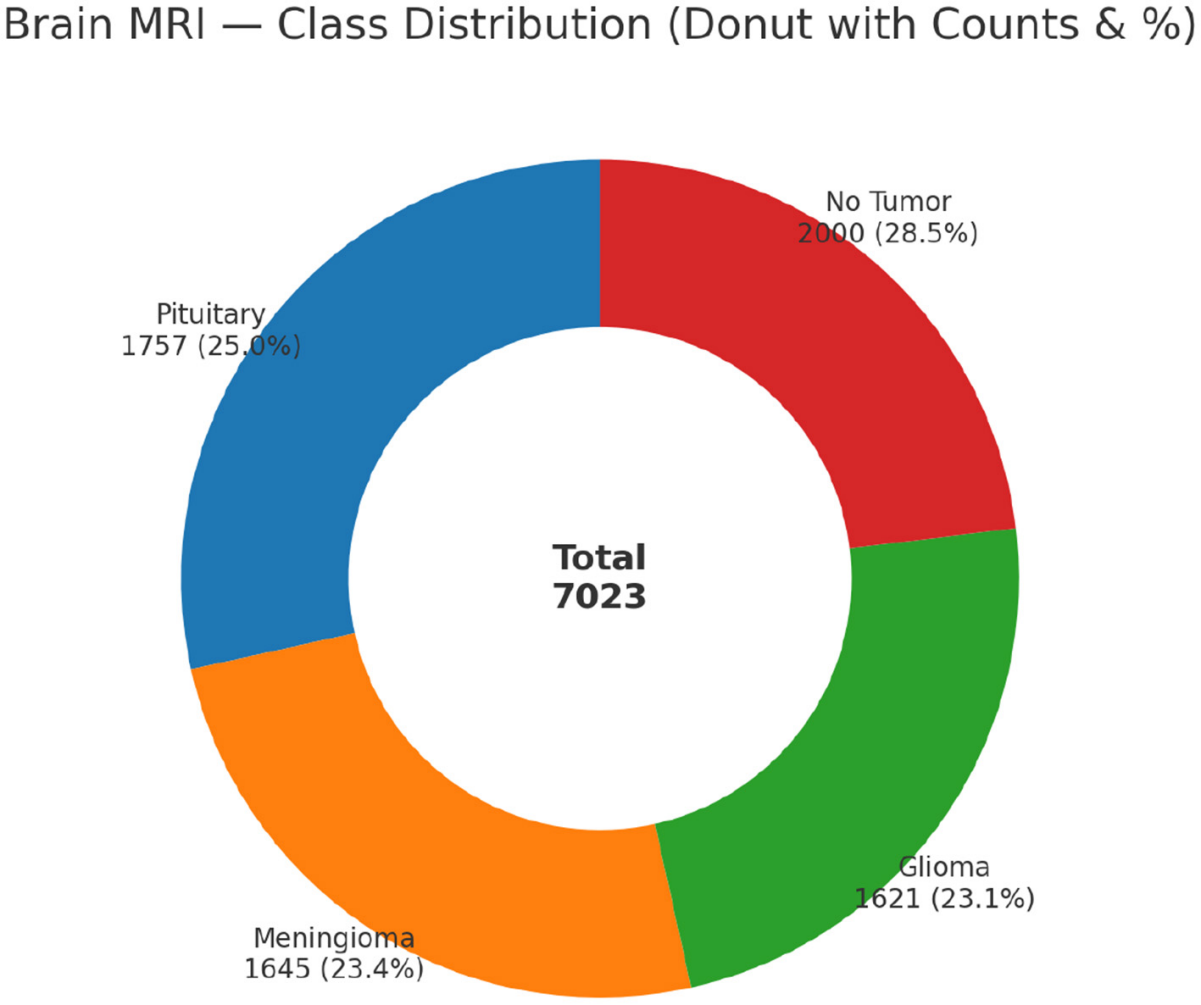
Class distribution of the dataset.

**Figure 2:**
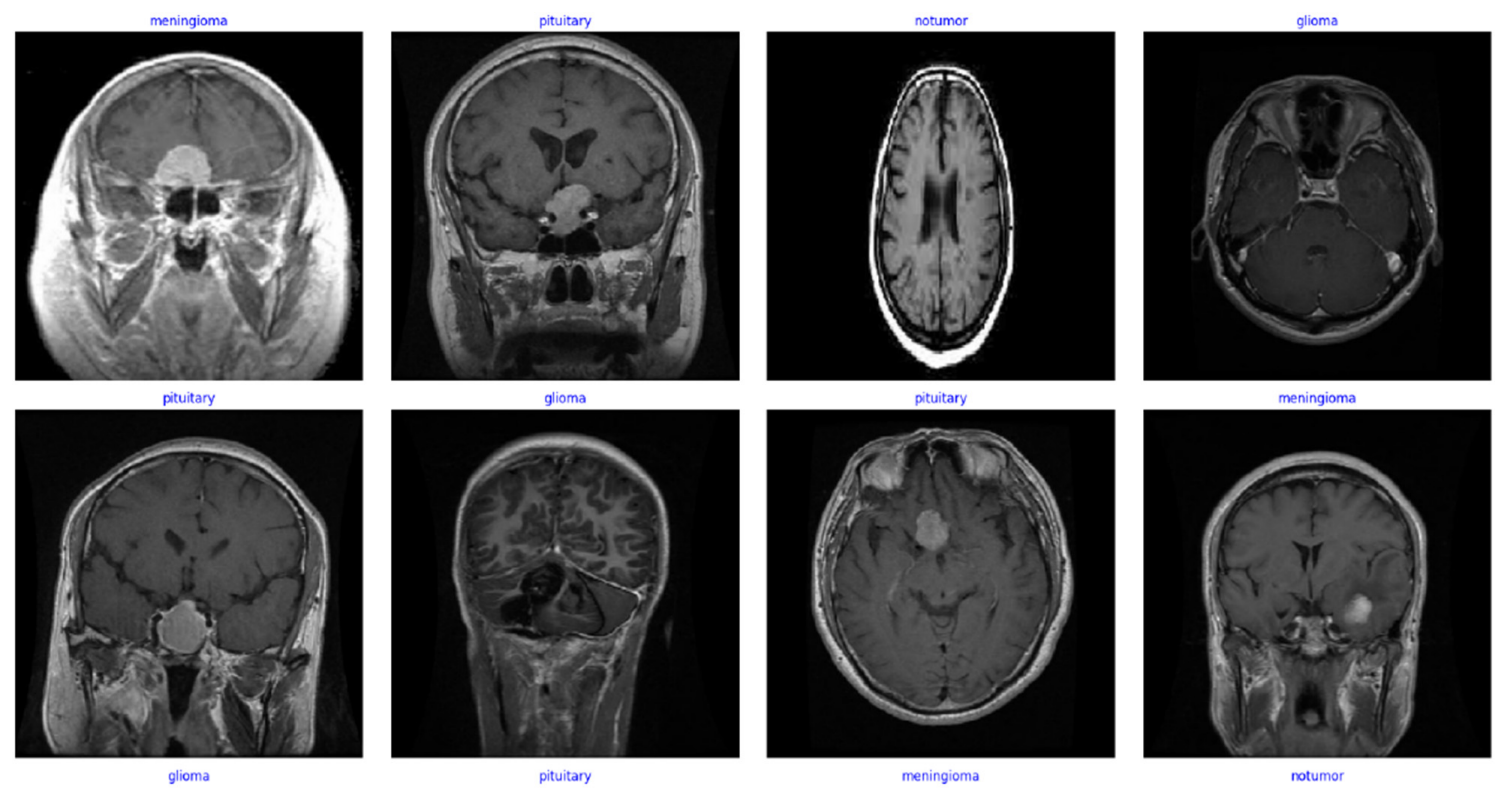
Representative preprocessed MRI samples from the heterogeneous tumor dataset.

The MRI image dataset used in this study is publicly available. The dataset includes a total of 7,023 MRI images. The images were organized into a training and a testing set. The dataset consists of brain MRI images classified into four categories: No Tumor, Pituitary, Meningioma, and Glioma in Figure 1. Each category contains tumors of different stages and sizes, adding variety to the dataset. This makes it more challenging for the deep learning model. It helped it learn to recognize small but important differences between tumor types (30). Figure 2 shows the balance distribution of the dataset. The balanced distribution ensures reliable training and evaluation of machine learning models.

### 3.2 Data preprocessing

We selected MRI images for the dataset based on two factors: clarity and usefulness. Each scan needed to clearly display the tumor's key features so that the deep learning model could learn effectively. Images that were blurry, incomplete, or not relevant to tumor analysis were removed. This careful selection process ensured the dataset stayed reliable and focused on high-quality samples. By keeping only the best images, we trained the models that can accurately recognize tumor patterns in real-world medical settings.

**Figure 3:**
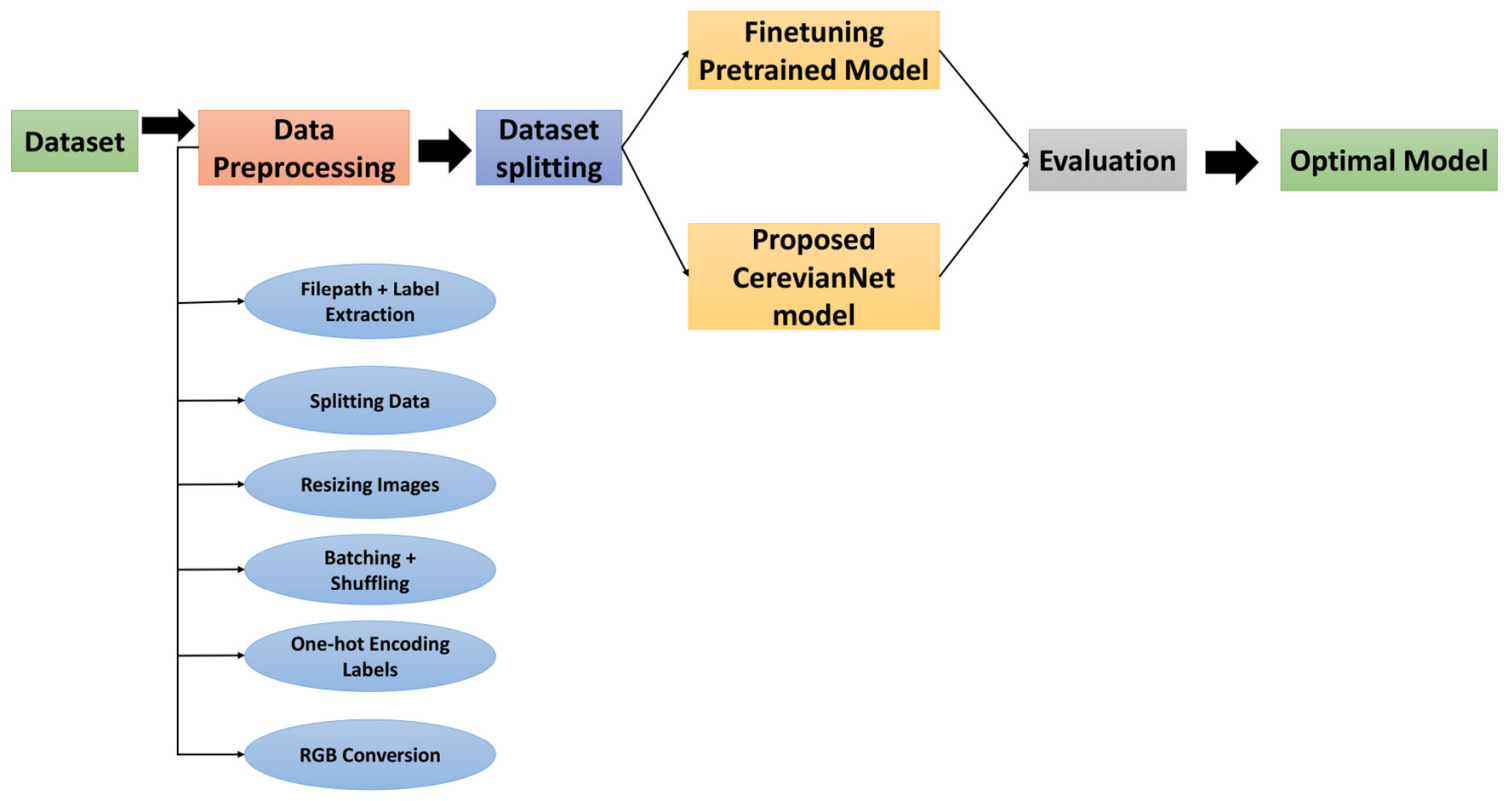
Workflow of our proposed system.

Several preprocessing steps were taken to achieve a better generalization in Figure 3. Grayscale images are computationally less complex to process than color images. This is crucial in medical image analysis, where large datasets are common (31). Each image was converted to grayscale using Equation 1. It simplified the data by reducing it to a single-channel image.


$$\begin{array}{l} G\left(x,y\right)=0.299\cdot R\left(x,y\right)+0.587\cdot G\left(x,y\right)+0.114\cdot B\left(x,y\right) \end{array}$$ (1)

Equation 2 was applied to reduce the noise in the grayscale images. It was a Gaussian filter. We applied a 3 × 3 kernel over the images. The blurring helps in smoothing the image and makes the edges more defined during subsequent steps.


$$\begin{array}{l} G_{s}\left(x,y\right)=G\left(x,y\right)*K \end{array}$$ (2)

where *K* is the Gaussian kernel of size 3 × 3, and * denotes the convolution operation.

The blurred image undergoes thresholding to segment it into binary form using Equation 3. A pixel intensity threshold of 45 is applied. This transformed all pixel values below this threshold to 0, while those above it became 255. This process created a binary image that highlights the regions of interest.


$$B(x,y)=\{\begin{array}{ll} 255, & \text{if }G_{s}(x,y)>T\\ 0, & \text{otherwise} \end{array}$$ (3)

where *T* = 45 is the threshold value.

Morphological operations, such as erosion and dilation, were applied using Equations 4, 5, sequentially. Erosion removes small noise by shrinking the white regions in the binary image. Dilation then restores the size of the significant features while retaining the removed noise.


$$\begin{array}{l} B_{e}\left(x,y\right)=E\left(B\left(x,y\right),S\right)\;\text{(Erosion)} \end{array}$$ (4)

$$\begin{array}{l} B_{d}\left(x,y\right)=D\left(B_{e}\left(x,y\right),S\right)\;\text{(Dilation)} \end{array}$$ (5)

Contours represent the boundaries of distinct objects within the image. From the identified contours, the largest contour is selected based on maximum area using Equation 6. The four extreme points of the largest contour are identified.


$$\begin{array}{l} C_{\text{max}}=\arg\underset{C_{i}}{\max}\text{Area}\left(C_{i}\right) \end{array}$$ (6)

where *C*_*i*_ represents the set of detected contours.

These are the leftmost point, the rightmost point, the topmost point, and the bottommost point. These points define a rectangular bounding box that encloses the main object in the image. Using the extreme points, the original image is cropped to retain only the main object using Equation 7.


$$\begin{array}{l} I_{\text{crop}}=I\left[P_{\text{top}}\left(y\right):P_{\text{bottom}}\left(y\right),P_{\text{left}}\left(x\right):P_{\text{right}}\left(x\right)\right] \end{array}$$ (7)

where *P*_top_, *P*_bottom_, *P*_left_, and *P*_right_ are the extreme points of the largest contour. This cropping removes unnecessary background regions, enhancing focus on the primary content.

To ensure uniformity in input size for all images, cropped images were resized to a fixed dimension of 256 × 256 pixels using Equation 8.


$$\begin{array}{l} I_{\text{resized}}\left(u,v\right)=I_{\text{crop}}\left(\frac{u}{256}\cdot H,\frac{v}{256}\cdot W\right) \end{array}$$ (8)

where *H* and *W* are the height and width of *I*_crop_.

All the images were normalized. Normalization ensures that all input features, like pixel intensities, are on a similar scale. This helps prevent issues like extremely small or large gradient values. This process made the learning process more stable and efficient (32).

Figure 2 illustrates the curated subset of preprocessed MRI images comprising distinct tumor classes. In this study, the classes shown in the sample images constitute core pathological categories. The displayed images highlight the effectiveness of the standardized preprocessing pipeline applied to raw medical data. The dataset emphasizes diversity in tumor morphology, with samples spanning varying stages.

In our code, the first split of the dataset occurs for the training dataset, where the training dataset is obtained from train_data_path, and the test dataset is sourced from the test_data_path. The test dataset is then further split into a validation and test dataset using a 50/50 split. Therefore, half of the data from the test set is used for validation, and the other half is used for testing. This split process is helpful in that the model is trained on the training set of the data, validated on a different piece of the training data to avoid overfitting, and then finally tested on the last piece of data. In terms of training configuration, the code does not utilize early stopping or learning rate scheduling. The model trains for 50 epochs and a constant learning rate of 0.001 using Adamax as the optimizer. Learning rate scheduling would have been useful if the validation accuracy had become stagnant, allowing the model to temporarily decrease the learning rate rather than maintain a constant one; however, this is not currently implemented.

This code does not incorporate data augmentation, which, in general, increases generalization capabilities by artificially expanding the training set size. The authors may have excluded augmentation to keep the model simpler or due to limited computing resources (data augmentation does increase computational requirements during training). In any case, the authors may have chosen not to augment the dataset (including rotations, flips, and zooming) that could plausibly have made the final model more robust, given that they only had 60 unique training data examples to work with.

The authors did not implement any of the techniques that reduce complexity in the model (depthwise separable convolutions, pruning, quantization). These models can be optimized for lower inference times and less memory on edge devices. The authors could have reduced complexity through the implementation of depthwise separable convolutions; however, they are not used in the model included in this architecture.

The architecture can be summarized through pseudocode or by using tf.keras.utils.plot_model. This architecture includes a series of convolutional blocks that all share a common trait with respect to Conv2D, BatchNormalization, MaxPooling, and Dropout layers. At the last convolution block, the features are flattened and sent to a few fully connected layers, and end with a softmax output for classification. Using LeakyReLU activation and L2 regularization enhances stability and robustness to overfitting.

### 3.3 Finetuning deep learning models

**Figure 4:**
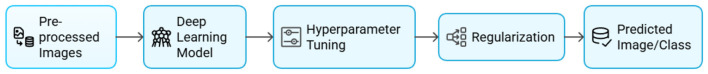
Fine-tuning deep learning models.

The adoption of fine-tuning strategies on pretrained deep learning architectures forms the methodological backbone of this study. This addressed the inherent challenges of limited annotated medical imaging data. We evaluated seven state-of-the-art models: EfficientNetB3, ResNet-50, ResNet-101, AlexNet, Inception V3, DenseNet121, and Swin Transformer. Figure 4 illustrates the fine-tuning procedure of deep learning models.

These models are selected for their proven efficacy in feature extraction across diverse domains. These architectures were initialized with weights pretrained on the ImageNet dataset. The models adapt generic visual representations to the subtle context of tumor morphology. This approach circumvents the need for training models from scratch, a critical advantage given the scarcity of large, annotated neuroimaging datasets. EfficientNetB3 leverages compound scaling to balance resolution, depth, and width efficiently, while Swin Transformer employs shifted-window self-attention to capture long-range spatial dependencies in high-resolution MRI slices. Such architectural diversity ensures robustness against variability in MRI acquisition protocols. The ResNet variants, with their residual connections, mitigate gradient degradation during training. Meanwhile, Xception and Inception V3 utilize multi-scale convolutional filters to isolate discriminative patterns across tumor subregions. DenseNet121, through its dense inter-layer connectivity, enhances feature reuse, which is critical for identifying overlapping pathological signatures in heterogeneous gliomas. By fine-tuning these models, we ensure they prioritize domain adaptation over memorization, thereby improving generalizability. This strategy not only reduces overfitting but also enhances the model's ability to generalize across various institutional imaging protocols.

### 3.4 Proposed model architecture

**Figure 5:**
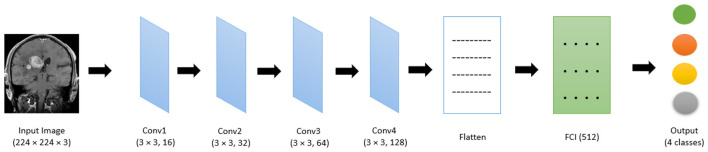
Architecture of our proposed system.

The proposed lightweight in Figure 5 convolutional neural network (CNN) is specifically designed for multi-class classification of medical image datasets. It consists of eight convolutional layers, grouped into four hierarchical blocks, followed by a dense classification head. The network is optimized to efficiently extract and process hierarchical features while minimizing computational costs. It is crucial for medical imaging tasks involving limited datasets.

Each convolutional block in the architecture contains two 3 × 3 convolutional layers with “same” padding to preserve spatial dimensions. The convolutional layers are followed by batch normalization, LeakyReLU activation (with α = 0.1), and a 2 × 2 max pooling layer. This arrangement was instrumental in progressively reducing the spatial dimensions of the feature maps while retaining essential information. The number of filters in each block doubles sequentially, starting from 16 in the first block and increasing to 128 in the fourth block (16 → 32 → 64 → 128). This progressive increase in the filter count enabled the network to capture both low-level features, such as edges and textures, and high-level features. It was critical for an accurate classification.

The use of batch normalization after each convolutional layer and dense layer ensures improved training stability and faster convergence by normalizing the input distributions to these layers. This not only accelerated the learning but also minimized the risk of vanishing or exploding gradients. It solves a common problematic situation in deeper networks. Additionally, the LeakyReLU activation function is employed to prevent the “dying ReLU” problem. It ensured that neurons in the network continue to learn even when they produce negative outputs. The LeakyReLU function, with α = 0.1, introduces a small slope for negative inputs. It provided a balance between non-linearity and gradient flow.

In our model, we use several techniques to improve performance and avoid overfitting which is mention in Algorithm 1. We'll use a dropout technique very broadly throughout the architecture to regularize the model. When a convolutional block is complete, we will apply a dropout of 25%, which means that during training, we are randomly dropping 25% of the neurons from those model layers. This strategy helps to avoid the model relying on certain neurons and helps to generalize better. After the large dense layers, we employ 50% dropout, which is not uncommon for fully connected layers and is commonly used to reduce overfitting, and very often with larger connected layers and many more parameters. We will employ L2 regularization in the weights of the dense layers. L2 works by applying a penalty term in the loss function for weights that are deemed to be too large. Thus, L2 helps avoid the fitting behavior we discussed as model bias. The regularization at 0.001 will keep the weights small, which makes the model more stable and helps avoid overfitting the model, mainly when we have ugly or useless features in the data.

Algorithm 1 Custom CNN architecture with pretrained model evaluation.


1: **Input:** dataset , pretrained_models {*M*_1_, *M*_2_, …, *M*_*n*_}, custom_model *C*
2: **Output:** best_model
3: **Step 1: Train and Evaluate Pretrained Models**
4: **for** *M*_*i*_∈{*M*_1_, *M*_2_, …, *M*_*n*_} **do**
5: Train *M*_*i*_ on _*train*_ {Train pretrained models}
6: Accuracy_*i*_←Validate(*M*_*i*_, _*val*_) {Validation performance}
7: **end for**
8: **Step 2: Train Custom CNN Model**
9: Train *C* on _*train*_ {Train custom architecture}
10: Accuracy_*C*_←Validate(*C*, _*val*_) {Validation performance of custom model}
11: **Step 3: Select the Best Model**
12: best_model←argmax{Accuracy_*i*_, Accuracy_*C*_} {Identify highest-performing model}
13: **Step 4: Fine-tuning the Best Model**
14: **if** best_model = *C* **then**
15: Fine-tune *C* with hyperparameter tuning
16: **else**
17: Fine-tune *M*_*i*_ {Refine pretrained model}
18: **end if**
19: **Return:** best_model

The Dense layers in your model are fully connected layers where every neuron in those layers is connected to the previous layers' neurons. The first dense layer has 128 neurons, and the second one has 64. Both dense layers utilize the LeakyReLU activation function, allowing for a little negative slope when the activation values are less than zero to prevent the –dying ReLU– problem when the neurons become inactive and no longer update. After the activation functions, batch normalization was used to stabilize the learning process, which helps ensure the activations are normalized and spread out more evenly. The output layer uses Softmax activation to output class probabilities in the multi-class case, where the output layer values add up to 1 since there is no more than 1 predicted class. This allows the model to predict the probability if the model predicted class 1 at 0.8.

After feature extraction, the flattened output from the final convolutional block is passed through a dense layer with 256 nodes. This fully connected layer applied batch normalization and LeakyReLU activation to consolidate the learned features into a lower dimensional representation. Later, it fed into a final softmax layer. The softmax layer converts the logits into a probability distribution, allowing the network to assign class probabilities to each input image. This step is essential for the multi-class classification of brain tumor categories.

The network is trained using the Adamax optimizer with a learning rate of 0.001, optimizing the categorical cross-entropy loss function. This combination ensures stable training dynamics and robust convergence, making the network suitable for medical imaging tasks. The model's lightweight nature, combined with its hierarchical feature extraction and efficient parameter utilization, provided an optimal balance between accuracy and computational efficiency. This design choice is particularly beneficial for scenarios involving limited computational resources or memory constraints, such as deploying the model on edge devices in healthcare settings.

### 3.5 Model configuration and hyperparameter

We will discuss the detailed specifications and hyperparameters of our proposed lightweight model CerevianNet, dedicated to Multi-Class Brain Tumor Classification, and the framework is based on TensorFlow/Keras. In this section, we summarize all model parameters and theoretical resource consumption:

- Layers:

– Convolutional layers:

* Four Conv2D layers (16, 32, 64, 128 filters) with kernel size (3, 3) and LeakyReLU activation function.
* Padding set to “same” for preserving input size.
* Batch normalization applied after each Conv2D layer for stabilization.

– Pooling:

* MaxPooling2D layers used (2 × 2 pool size) to reduce spatial dimensions.

– Dropout:

* Dropout rate of 0.25 after convolutional blocks to prevent overfitting.
* Dropout of 0.5 after dense layers.

– Dense layers:

* 128 units with LeakyReLU activation and L2 regularization =0.001.
* 64 units with the same activation function and regularization.
* Final dense layer with units equal to the number of classes, using softmax activation for multi-class classification.

- Model architecture:

– Input shape: (150, 150, 3) (for RGB images of size 150 × 150)
– Output: a softmax output layer with the number of units equal to the number of classes.

- Optimization:

– Optimizer: we choose Adamax for its stable convergence and regularization properties.
– Learning rate: we set the learning rate to 0.001.

- Loss function: we choose the Categorical Cross-Entropy because it is suitable for multi-class classification problems.
- Matrics: the model tracks accuracy to evaluate performance during training and testing.
- L2 regularization: applied to Dense layers (128, 64 units) with a regularization term 0.001 to reduce overfitting.
- Custom Callback (TestAccuracyCallback): evaluates test accuracy at the end of each epoch to monitor performance on the test set.
- Data Flow:

– For both training and testing, uses standard flow from dataframes.
– Data augmentation is not applied here, but can be added to improve generalization.

- Training specifications:

– Batch size: we set the Batch Size at 32.
– Epochs: initially, we use 50 epochs for training.
– Training method: model.fit() is used for training with training and validation data.

- Evaluation:

– Training accuracy & loss
– Validation accuracy & loss
– Test accuracy & loss
– Confusion matrix

## 4 Experimentation and results

**Figure 6:**
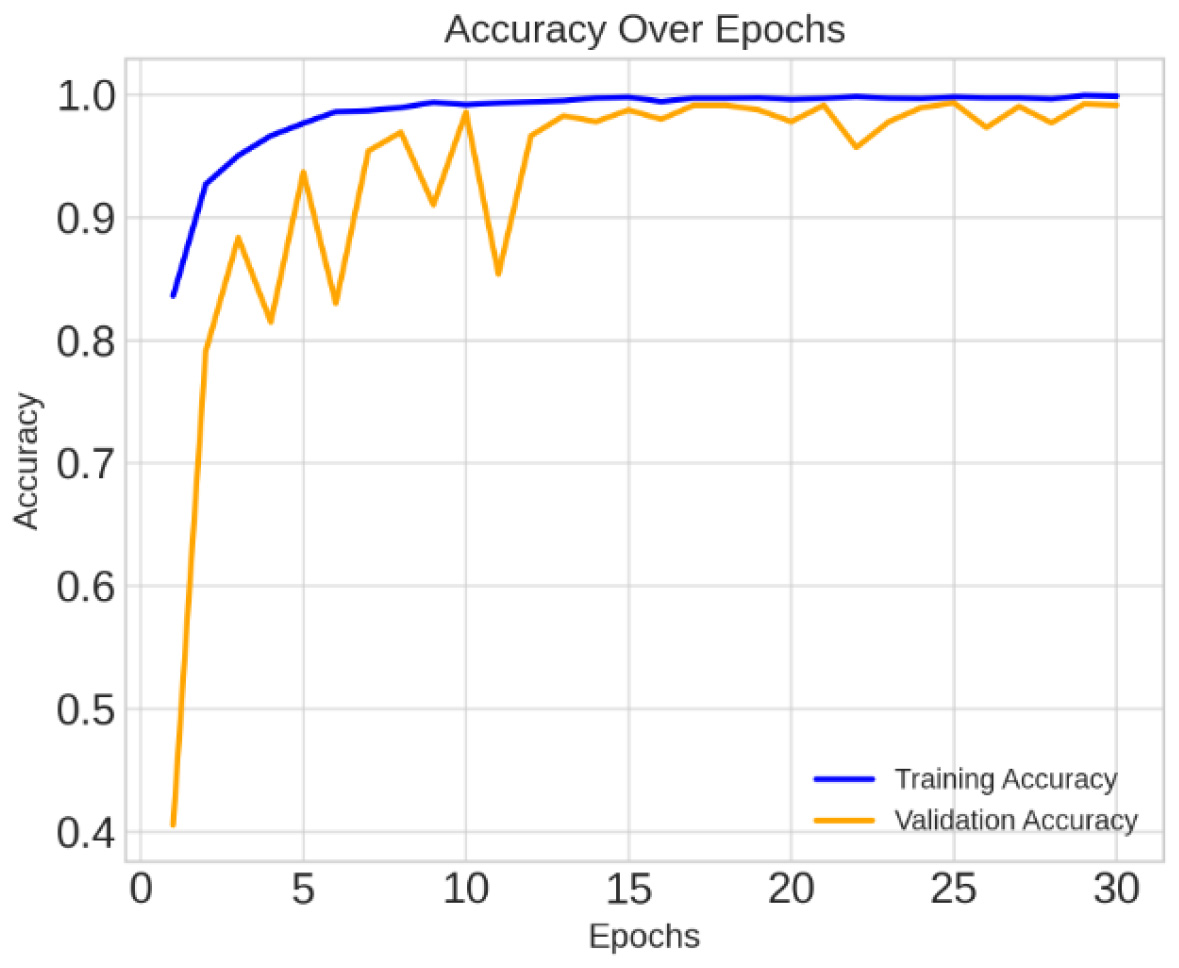
Training and validation accuracy curve of CerevianNet for dataset-1.

This section presents an in-depth analysis of the performance of our proposed lightweight CerevianNet model, alongside several state-of-the-art models for the classification of brain tumors using MRI images. We conducted a rigorous evaluation to assess the effectiveness of each model. Our experimentation spanned a diverse range of architectures. In this process, we used computationally efficient models suitable for resource-constrained environments to more complex and high-performing models. The results highlight the trade-offs between accuracy and computational efficiency, particularly emphasizing the practical advantages of our custom lightweight CerevianNet model and the enhanced accuracy in Figure 6.

The CerevianNet model demonstrates a high level of accuracy in classifying brain tumors, achieving an impressive overall accuracy of 98%. The training accuracy quickly converges to near-perfect levels within the first few epochs. It indicates the model's ability to learn effectively from the training data. Notably, the validation accuracy closely tracks the training accuracy, suggesting that the model is generalizing well to unseen data and is not overfitting. This is further supported by the consistent performance across epochs, with the model maintaining high accuracy throughout the training process.

Figure 6 provides a visual representation of the CerevianNet model's accuracy over epochs. The graph clearly shows the rapid convergence of both training and validation accuracy, highlighting the model's efficiency and robustness. The stable performance across epochs indicates that the model has learned a strong representation of the data and is capable of consistently accurate classification.

**Figure 7:**
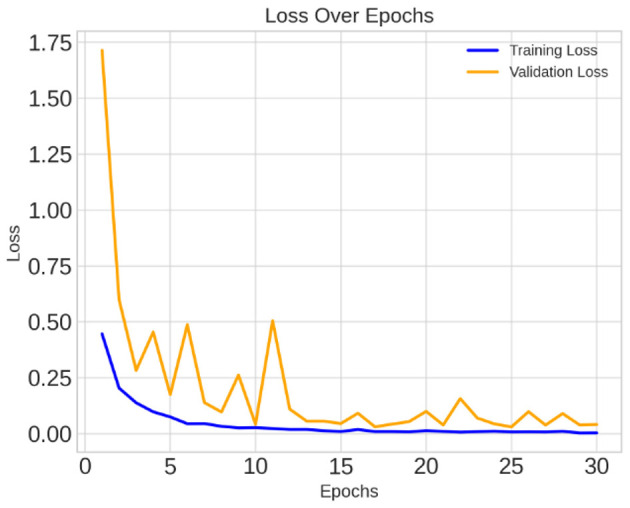
Training and validation loss curve of CerevianNet for dataset-1.

The loss curve in Figure 7 further demonstrates the CerevianNet model's robust learning and generalization capabilities. The training loss rapidly decreases during the initial epochs. This indicates the model's ability to quickly adapt to the training data. Moreover, the validation loss also exhibits a steady decline, closely tracking the training loss. Thus suggested that the model is learning a generalizable representation of the data and not overfitting. The consistent decrease in both training and validation loss across epochs indicates that the model was effectively minimizing the error and improving its predictive power.

**Figure 8:**
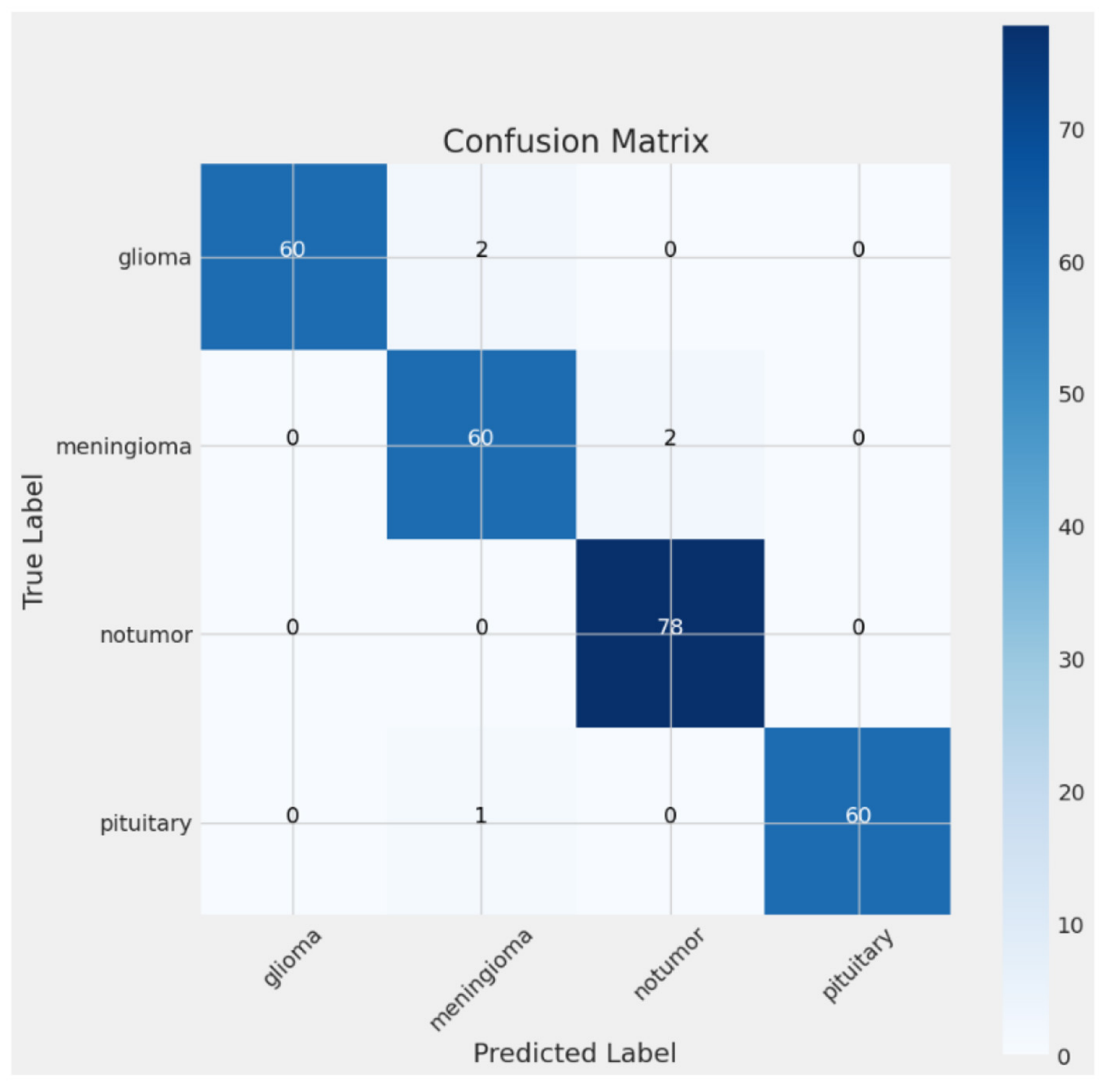
Confusion matrix of CerevianNet for dataset-1.

The confusion matrix presented in Figure 8 illustrates the CerevianNet model's robust performance across various brain tumor classifications. It visually represents the model's predictions against the true labels, precisely highlighting both correct classifications and the few instances of misclassification. The prominent diagonal pattern within the matrix signifies a high rate of correct classifications for all tumor types examined: glioma, meningioma, non-tumor, and pituitary. This indicates the model's overall effectiveness in accurately distinguishing between these categories.

While the model demonstrates high accuracy overall, there are a few instances of misclassification. Notably, there's a minor degree of confusion between glioma and meningioma. Two glioma cases are being misclassified as meningioma, and two meningioma cases are being misclassified as glioma. This slight overlap could be attributed to shared imaging characteristics between these two tumor types. A single pituitary case was misclassified as a meningioma. These small numbers of misclassifications underscore the model's general accuracy and its potential for practical use. This detailed examination not only highlights the model's strengths in differentiating between tumor types but also pinpoints areas where further refinement could potentially improve its diagnostic capabilities and reduce the observed confusion between glioma and meningioma.

**Figure 9:**
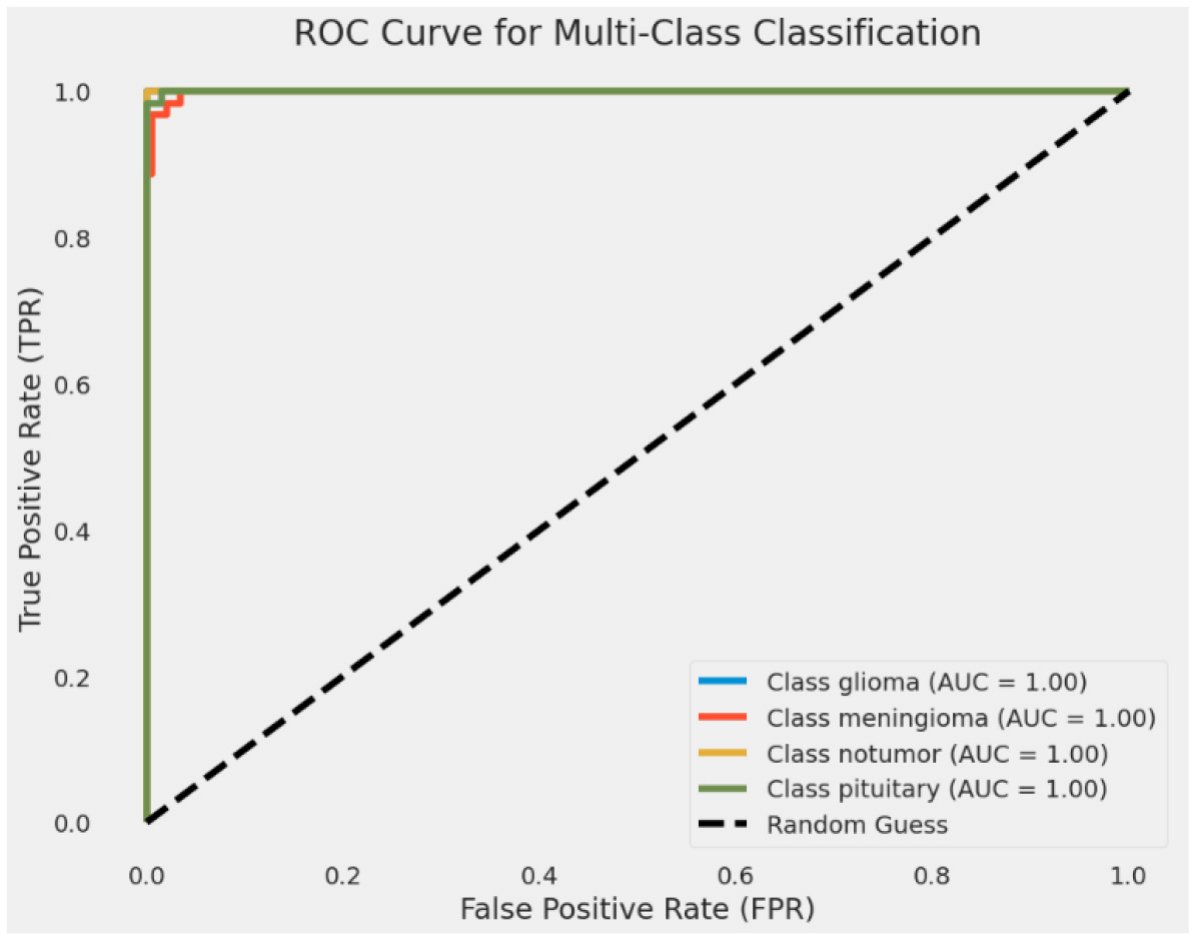
ROC-AUC curve of CerevianNet for Dataset-1.

The receiver operating characteristic (ROC) curves and area under the curve (AUC) values shown in Figure 9 provide a comprehensive evaluation of the model's performance for multi-class classification. The AUC values for all four classes—glioma, meningioma, non-tumor, and pituitary—are 1.00. This indicates that the model achieved perfect discrimination for all categories. These results highlight the model's exceptional ability to correctly classify each tumor type without errors. The ROC curve in the figure demonstrates the model's diagnostic accuracy and its potential effectiveness in real-world medical applications.

**Figure 10:**
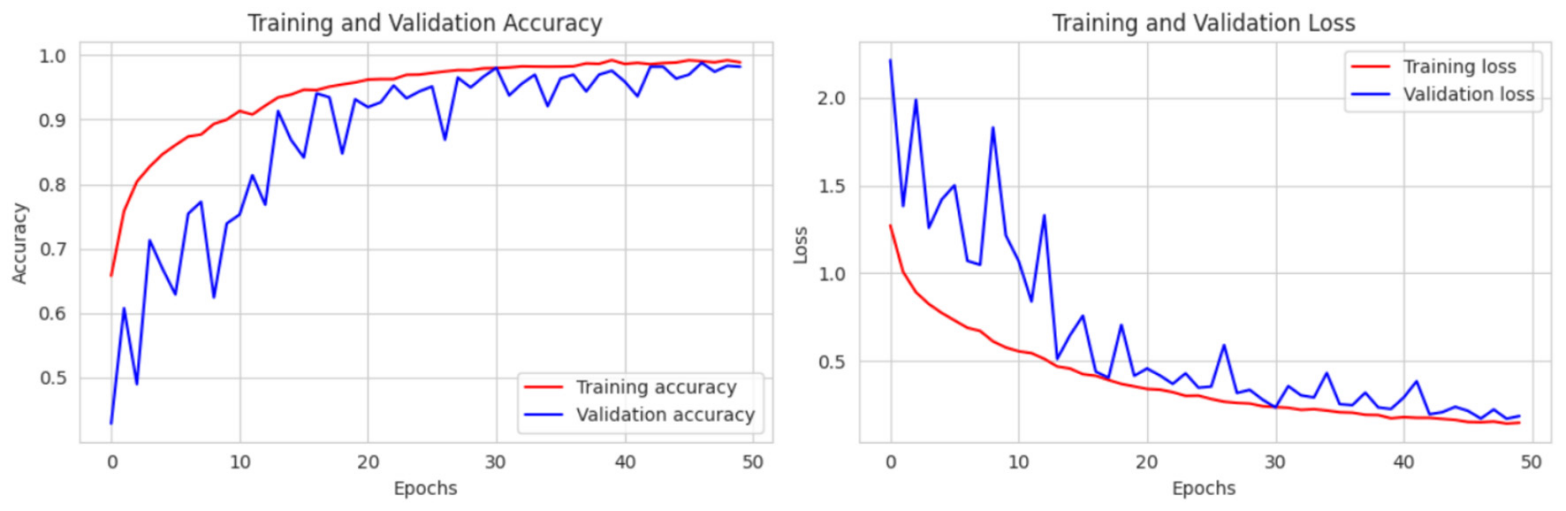
Training and validation accuracy and loss curve of CerevianNet for Dataset-2.

For another dataset from Kaggle with 7,023 images in four classes, we try the same batch size, learning rate, and 50 epochs as before and after training our model, in Figure 10 we achieved a training accuracy of 99.92% with a training loss of 0.117, a validation accuracy of 98.16% with a validation loss of 0.186 and finally after testing, achieved a testing accuracy of 97.1% with a testing loss of 0.22.

**Figure 11:**
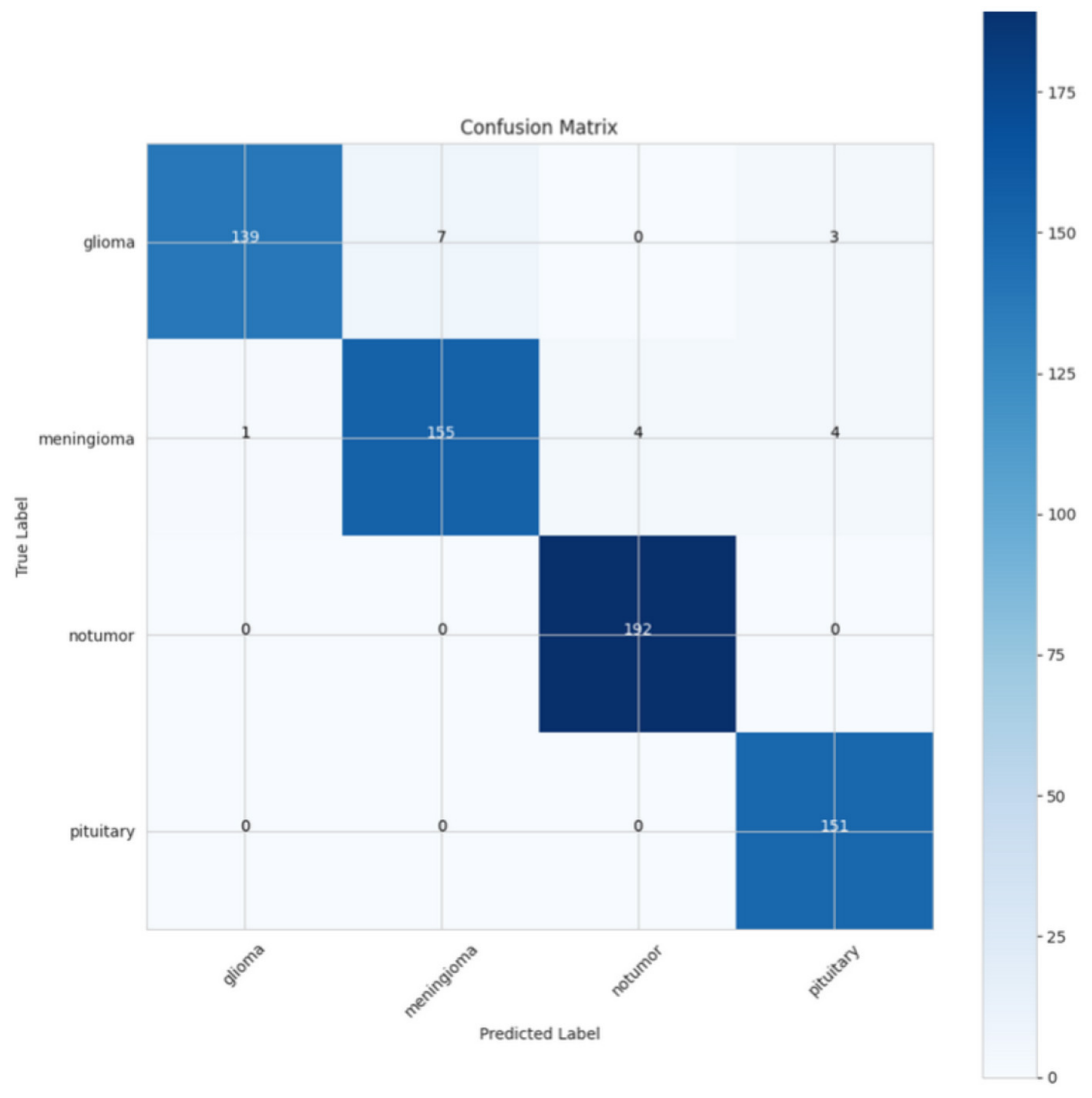
Confusion matrix of CerevianNet for dataset-2.

Figure 11 presents a confusion matrix that summarizes the model's classification capabilities on predicting the brain tumor types glioma, meningioma, non-tumor, and pituitary. The glioma type had 119 correctly classified observations, while seven of them were incorrectly classified as meningiomas and 3 were incorrectly classified as pituitary tumors. There were no observations incorrectly classified into the non-tumor type. Meningioma had 155 correctly classified observations, and the model exhibited minimal confusion, with 1 classifying as glioma, 4 as a non-tumor, and 4 as pituitary. The non-tumor type had excellent accuracy, predicting 192 correct observations and not including any predictions in other categories. As Meningioma and non-tumor were both accurately predicted across 151 observations, there were no prediction errors as well. The confusion matrices indicate that the model had a solid understanding of the data and was able to classify across cases with little confusion between glioma and meningioma types, when non-tumor and Pituitary predicted clear indicators of accuracy in the data.

**Figure 12:**
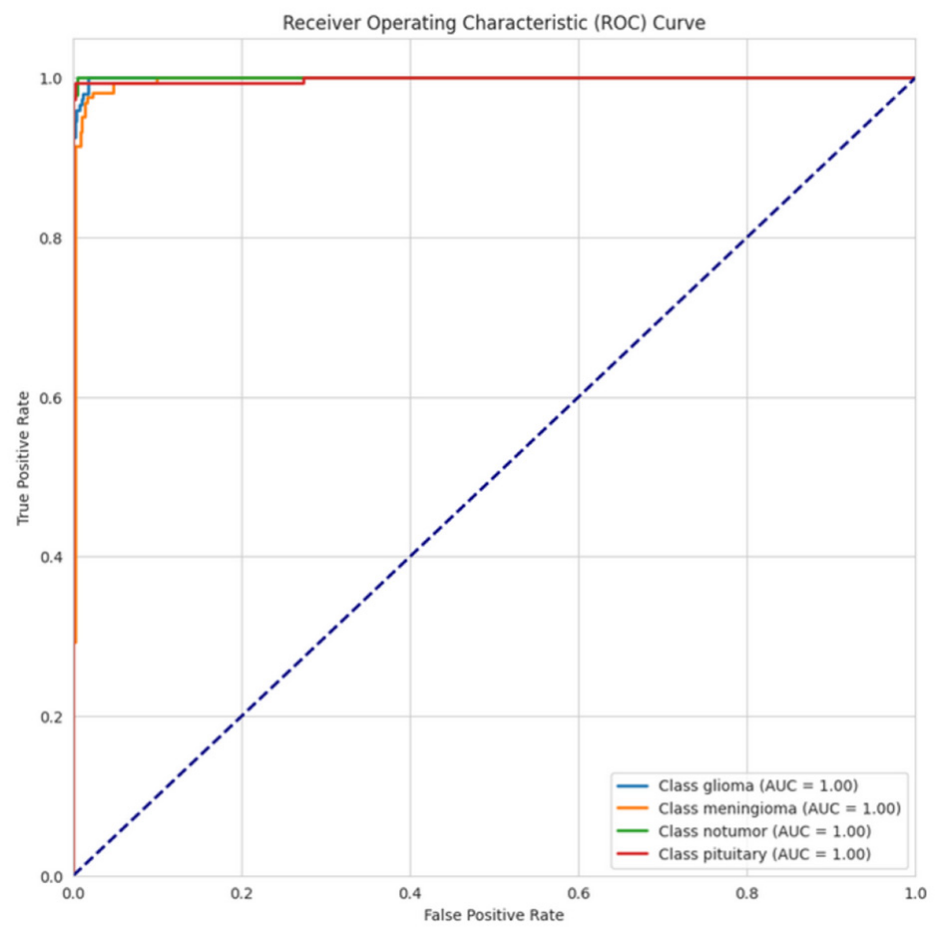
ROC-AUC curve of CerevianNet for dataset-2.

The ROC curve in Figure 12 illustrates the performance of a multi-class classification model, where each class—glioma, meningioma, non-tumor, and pituitary—has an ideal AUC of 1.00, indicating perfect classification. The curves are far above the diagonal baseline, indicating the model's excellent ability to differentiate between all classes.

**Figure 13:**
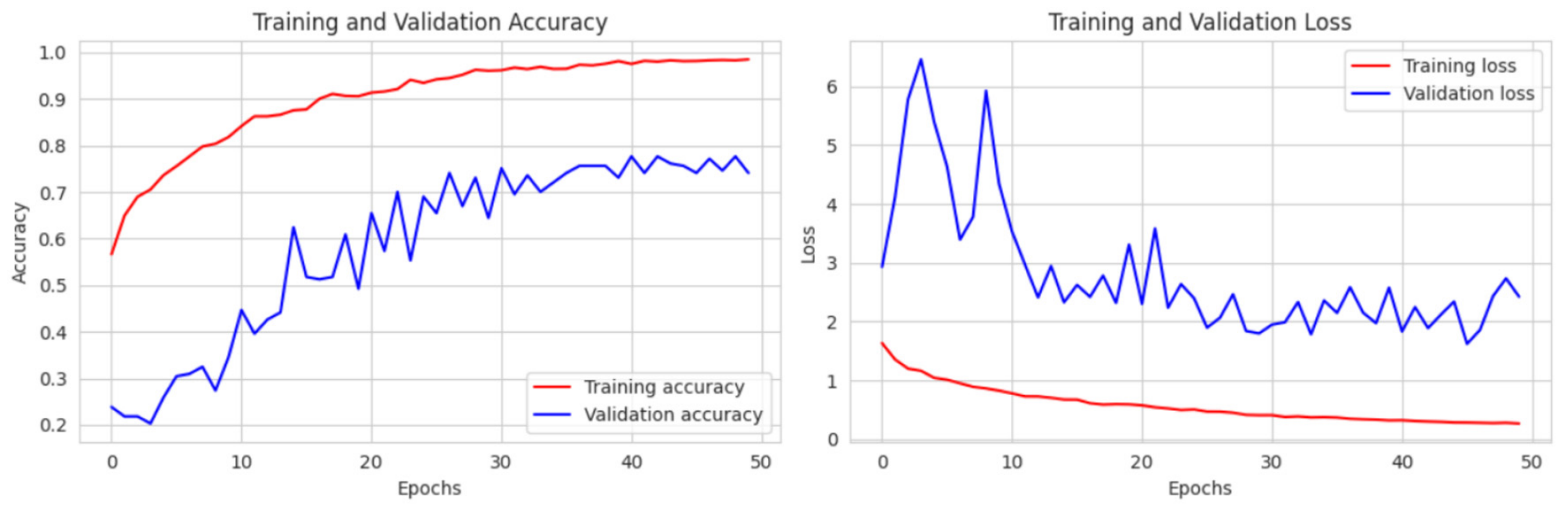
Training and validation accuracy and loss curve of CerevianNet for Dataset-3.

For another dataset from Kaggle with 3,264 images in 4 classes, we try the same batch size, learning rate, and 50 epochs as before and after training our model, in Figure 13 we achieved a training accuracy of 99.99% with a training loss of 0.214, a validation accuracy of 74.11% with a validation loss of 2.422 and finally after testing, achieved a testing accuracy of 75.63% with a testing loss of 2.61.

**Figure 14:**
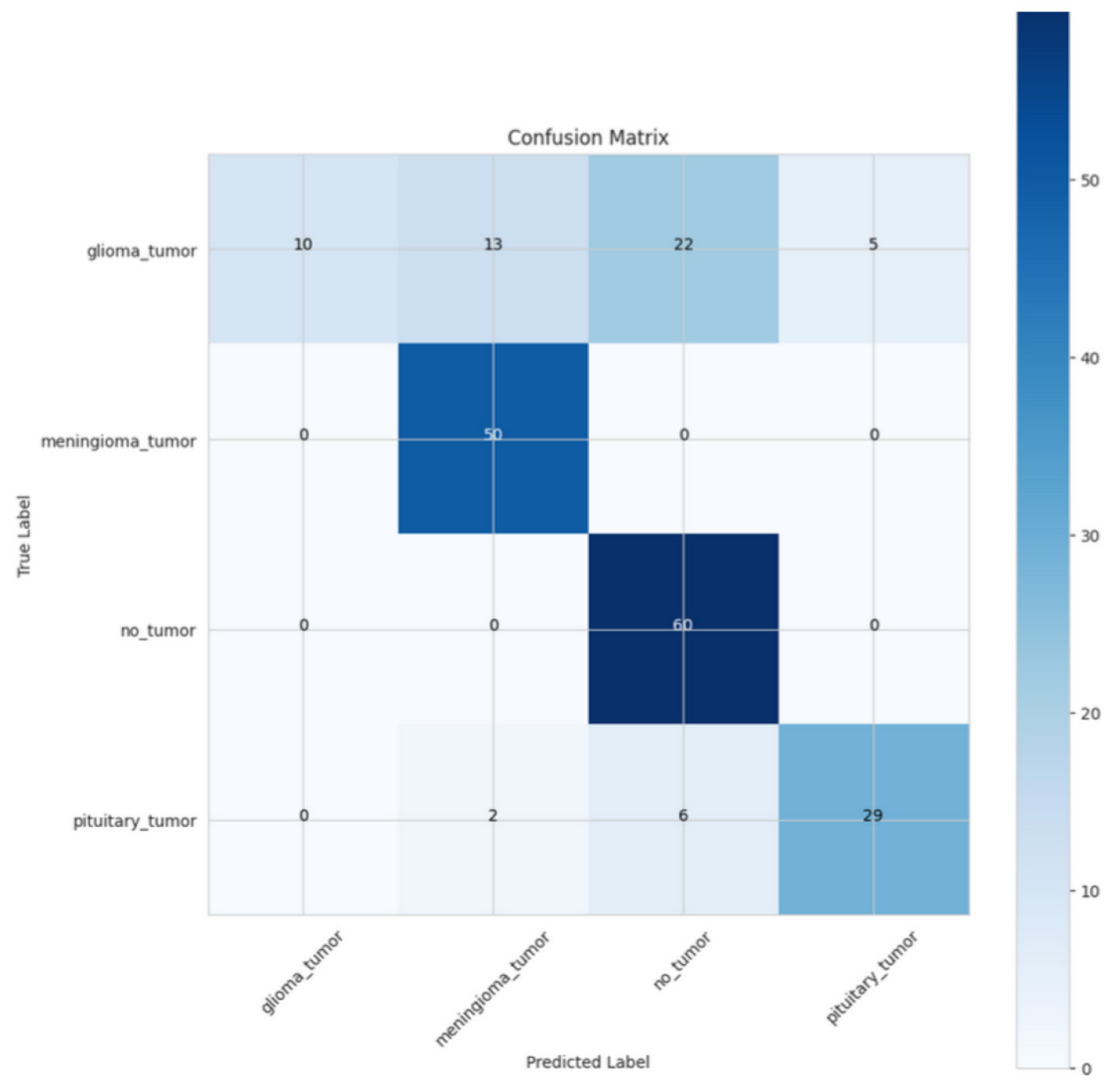
Confusion matrix of CerevianNet for Dataset-3.

The confusion matrix in Figure 14 shows the model's performance in categorizing four categories: glioma tumor, meningioma tumor, no tumor, and pituitary tumor. Altogether, the glioma tumor class markets a true positive with 10 correctly predicted cases; however, 13 cases are incorrectly predicted as meningioma tumors, 22 cases are predicted as no tumor, and five cases are predicted as pituitary tumors. The meningioma tumor class shows that we achieved an impressive accuracy rate with 50 correct predictions, and no predicted data points were incorrectly assigned to either of the other categories. The third class of no tumor showed we had a great performance with 60 correct predictions, and none were issued, indicating that the model can detect instances where there is no tumor. Finally, we can see in the pituitary tumor class that the model does predict 29 correctly, but makes some mistakes with two instances classified as meningioma tumors and six classified as no tumors. Overall, the model gives a fairly good general performance across the four classes; however, there is an issue in the glioma tumor category, where there are significant misclassifications, particularly in the no tumor and pituitary tumor categories. On the positive side, the meningioma tumor and no tumor categories had really high performance with minimal confusion with each other. The pituitary tumor class does have a few misclassifications, yet still shows generally good performance of being able to predict the class.

**Figure 15:**
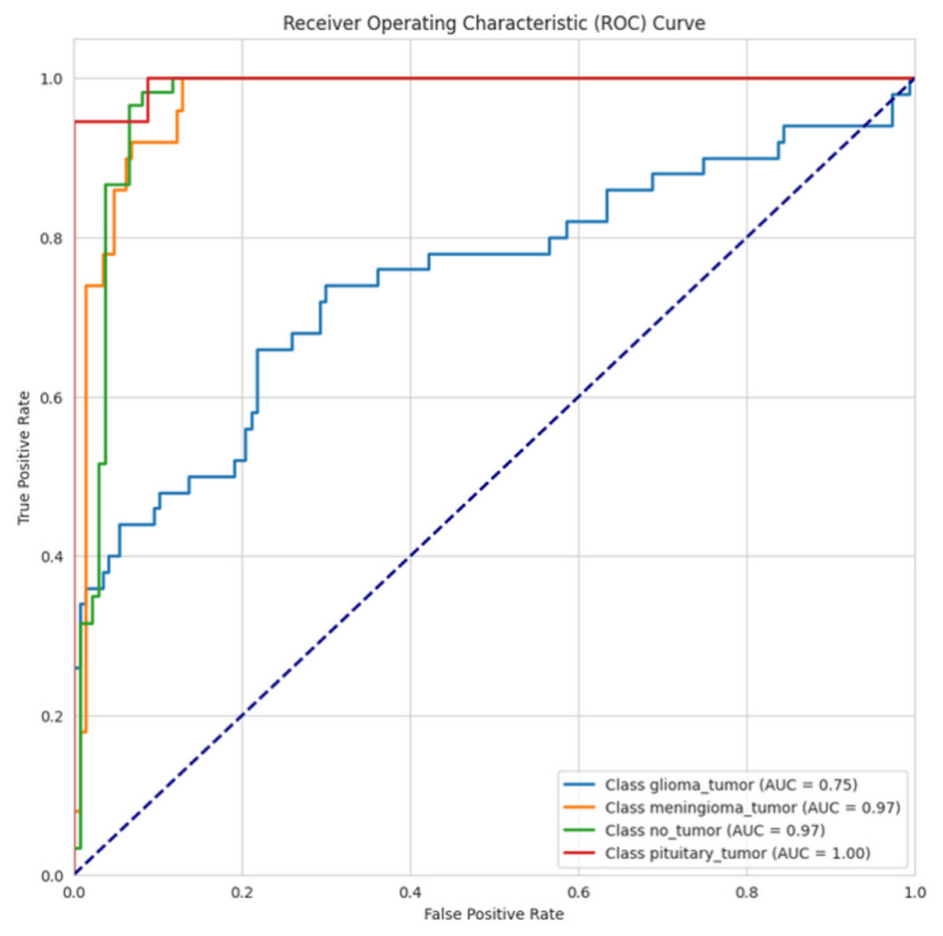
ROC-AUC curve of CerevianNet for Dataset-3.

Illustrated in Figure 15, the ROC curve depicts the results for a multi-class classification model with four classes: glioma_tumor, meningioma_tumor, no_tumor, and pituitary_tumor. The pituitary_tumor classification experienced perfect classification, with an optimal shape of (AUC = 1.00), and therefore it is not surprising. Meningioma_tumor and no_tumor classifications achieved the least, with an AUC of 0.97, which provides for strong classification results. However, the glioma_tumor classification returned a lower AUC of 0.75, displaying classification performance lower than the other groups.

**Figure 16:**
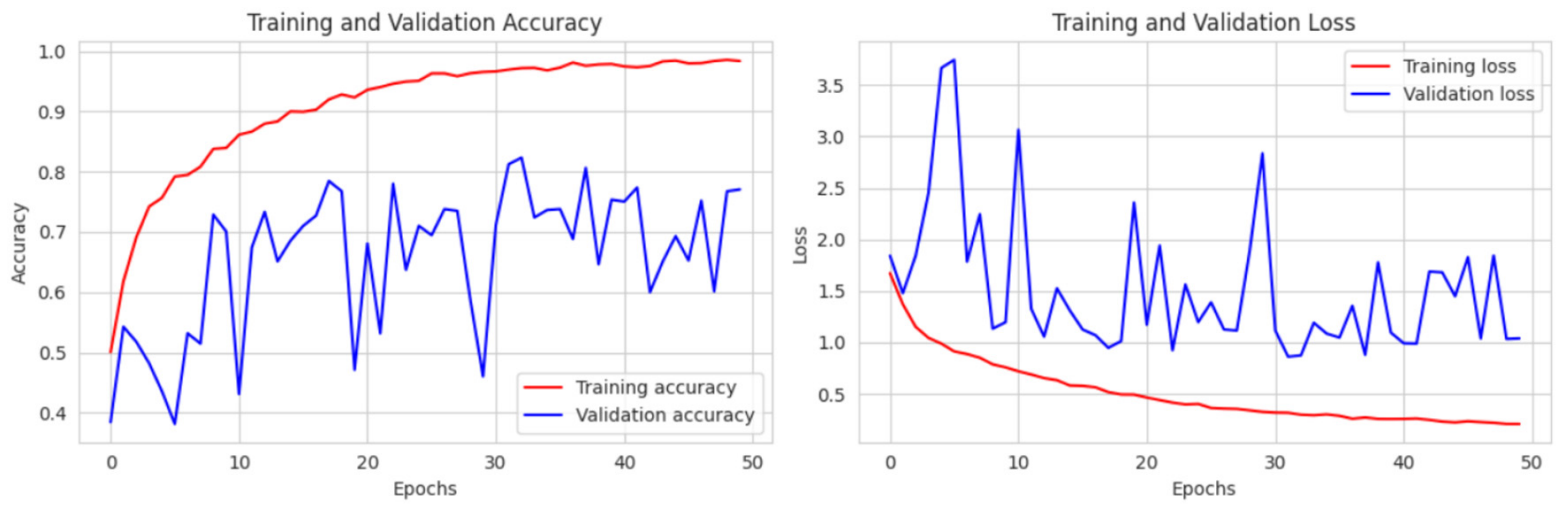
Training and validation accuracy and loss curve of CerevianNet for Dataset-4.1 + 4.2.

For this testing we merge another two dataset from Kaggle 4 classes, we try the same batch size, learning rate, and 50 epochs as before and after training our model, in Figure 16 we achieved a training accuracy of 99.34% with a training loss of 0.178, a validation accuracy of 74.05% with a validation loss of 1.039 and finally after testing, achieved a testing accuracy of 76.58% with a testing loss of 1.08.

**Figure 17:**
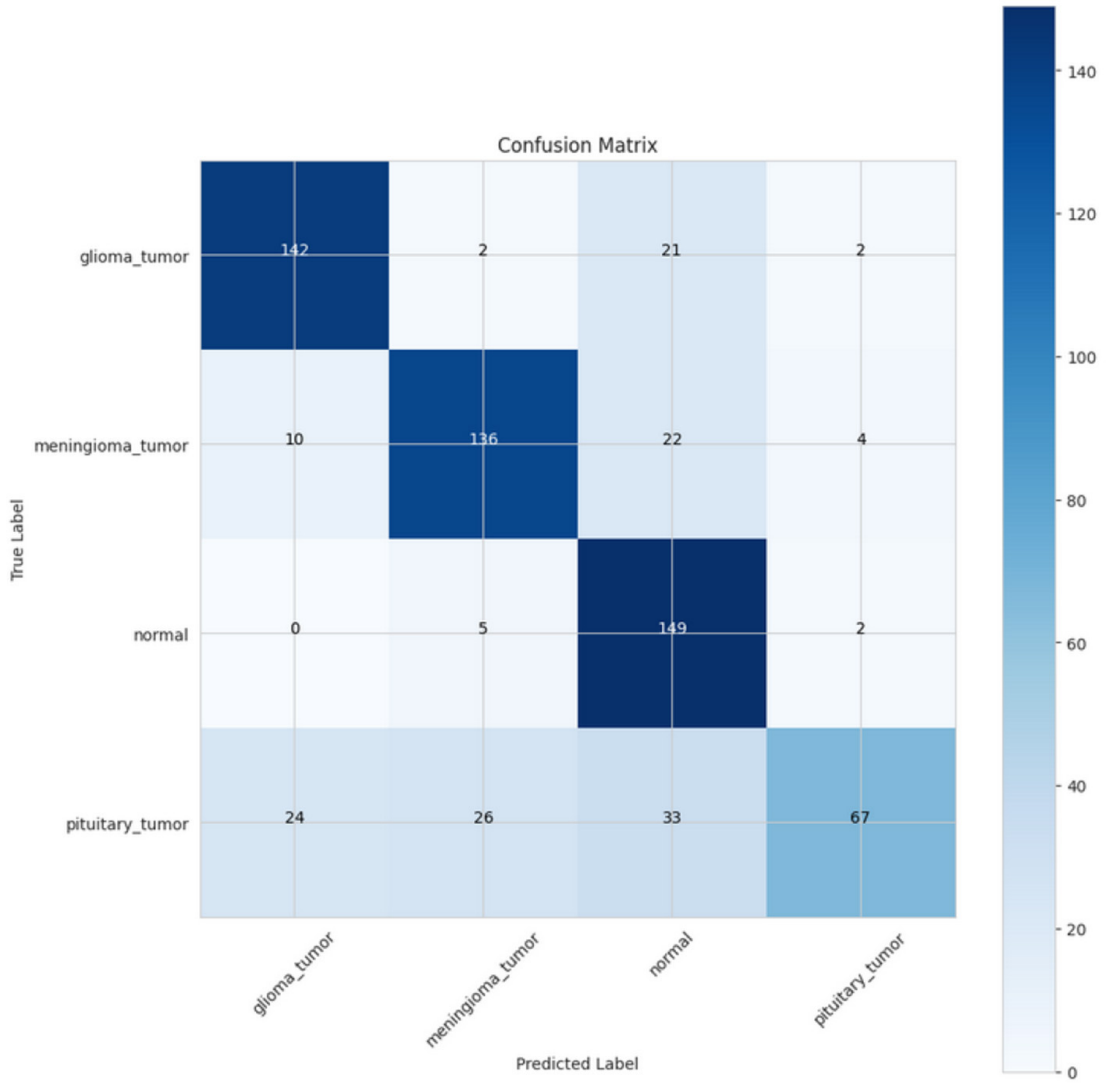
Confusion matrix of CerevianNet for Dataset-4.1 + 4.2.

The confusion matrix presented in Figure 17 provides a more comprehensive evaluation of the model's performance on training and testing datasets in the four categories: glioma tumor, meningioma tumor, normal (there was no tumor), and pituitary tumor. In terms of the glioma tumor category, 142 instances were correctly classified in this category; however, two were misclassified as meningioma tumors, 21 as normal, and two as pituitary tumors. For the meningioma tumor category, it was accurately predicted a total of 136 times, while being misclassified into glioma tumors 10 times, as normal 22 times, and as pituitary tumors four times. The normal category performed well with 149 total correct predictions and close to non-misclassifications into meningioma tumors (five times) and pituitary tumors (two times). Finally, the pituitary tumor class had a total of 67 correct predictions, with misclassifications into glioma tumors 24 times, meningioma tumors 26 times, and 33 times into the normal category. Overall, the model predicts equally well, but certainly, the glioma tumor and normal category performed better, showing quite high accuracies with generally very few misclassifications. However, there appears to be confusion with pituitary tumors juxtaposed with glioma tumors and meningioma tumors. Nevertheless, the overall prediction performance is good for separating the tumor categories from normal, suggesting a reasonably good degree of accuracy and reliability for all classes.

**Figure 18:**
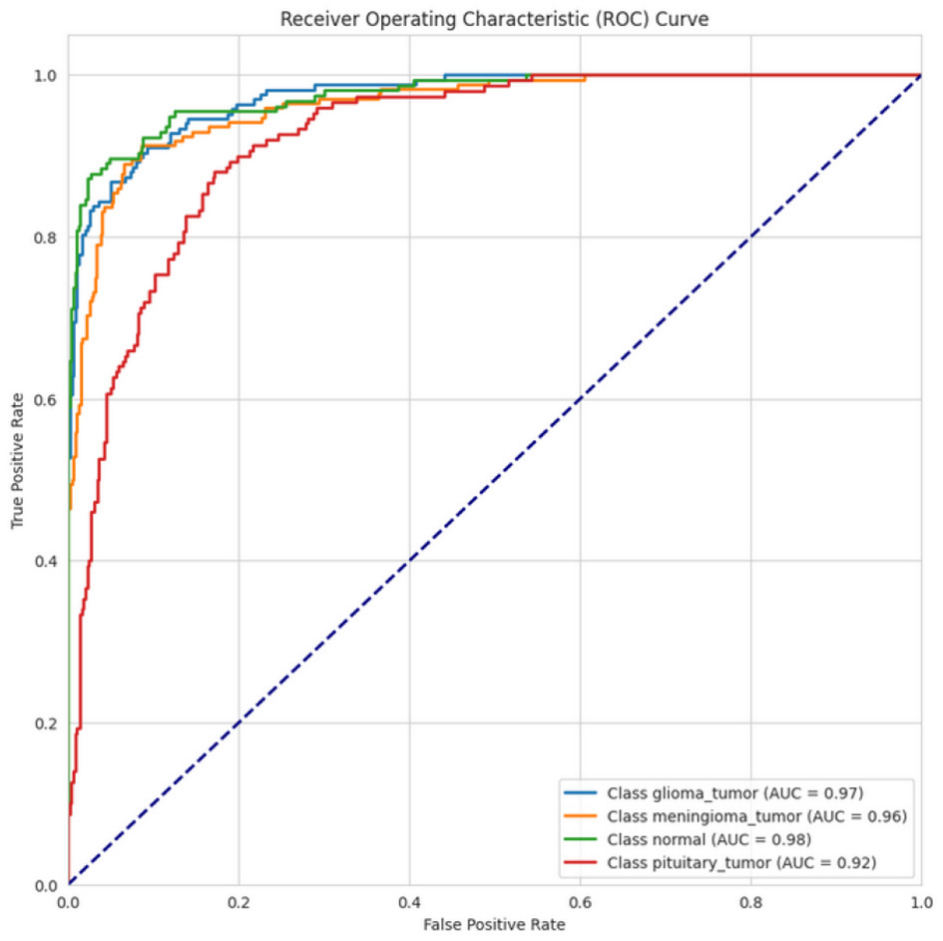
ROC-AUC curve of CerevianNet for Dataset-4.1 + 4.2.

The ROC curve depicted in Figure 18 illustrates the performance of four classes: glioma_tumor, meningioma_tumor, normal, and pituitary_tumor. The AUC of the normal class was the highest at 0.98, followed closely by glioma_tumor (AUC = 0.97), and meningioma_tumor (AUC = 0.96), with all classes exhibiting strong classification potential. Pituitary_tumor had a somewhat lower AUC at 0.92, implying somewhat weaker performance than the other classes.

**Figure 19:**
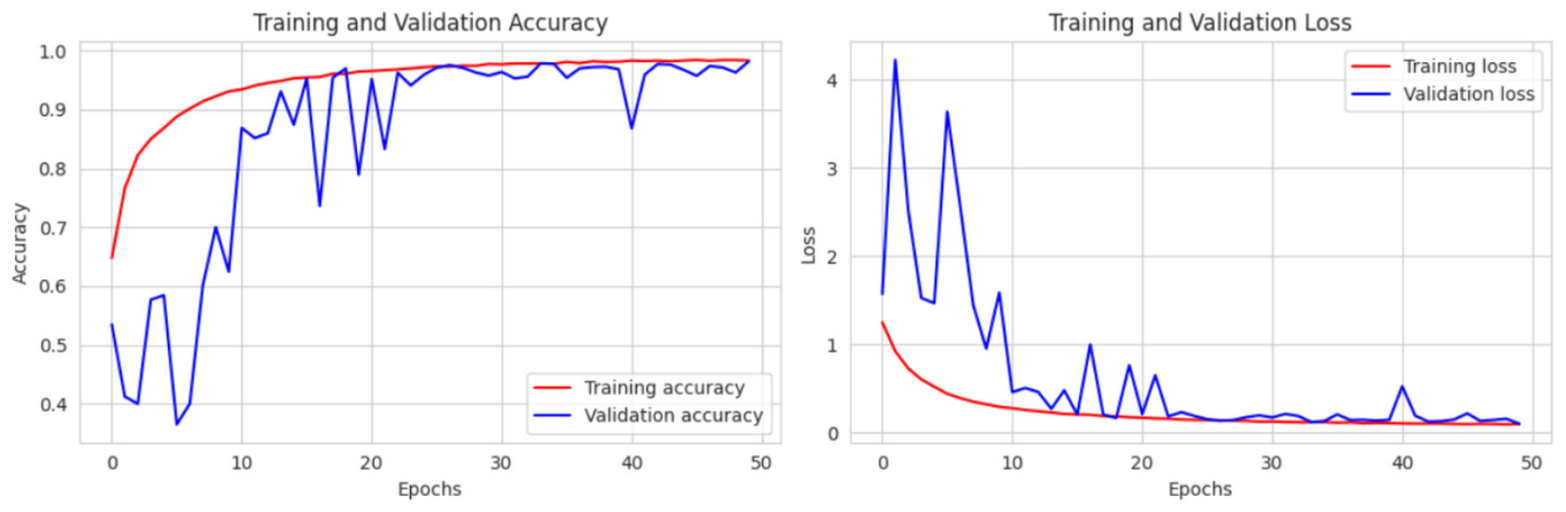
Training and validation accuracy and loss curve of CerevianNet for Dataset-5.

For this testing, we merge another two dataset from Kaggle which have 40,000 images with four classes, we try the same batch size, learning rate, and 50 epochs as before and after training our model, in Figure 19, we achieved a training accuracy of 99.67% with a training loss of 0.057, a validation accuracy of 98.17% with a validation loss of 0.102 and finally after testing, achieved a testing accuracy of 98.30% with a testing loss of 0.111.

**Figure 20:**
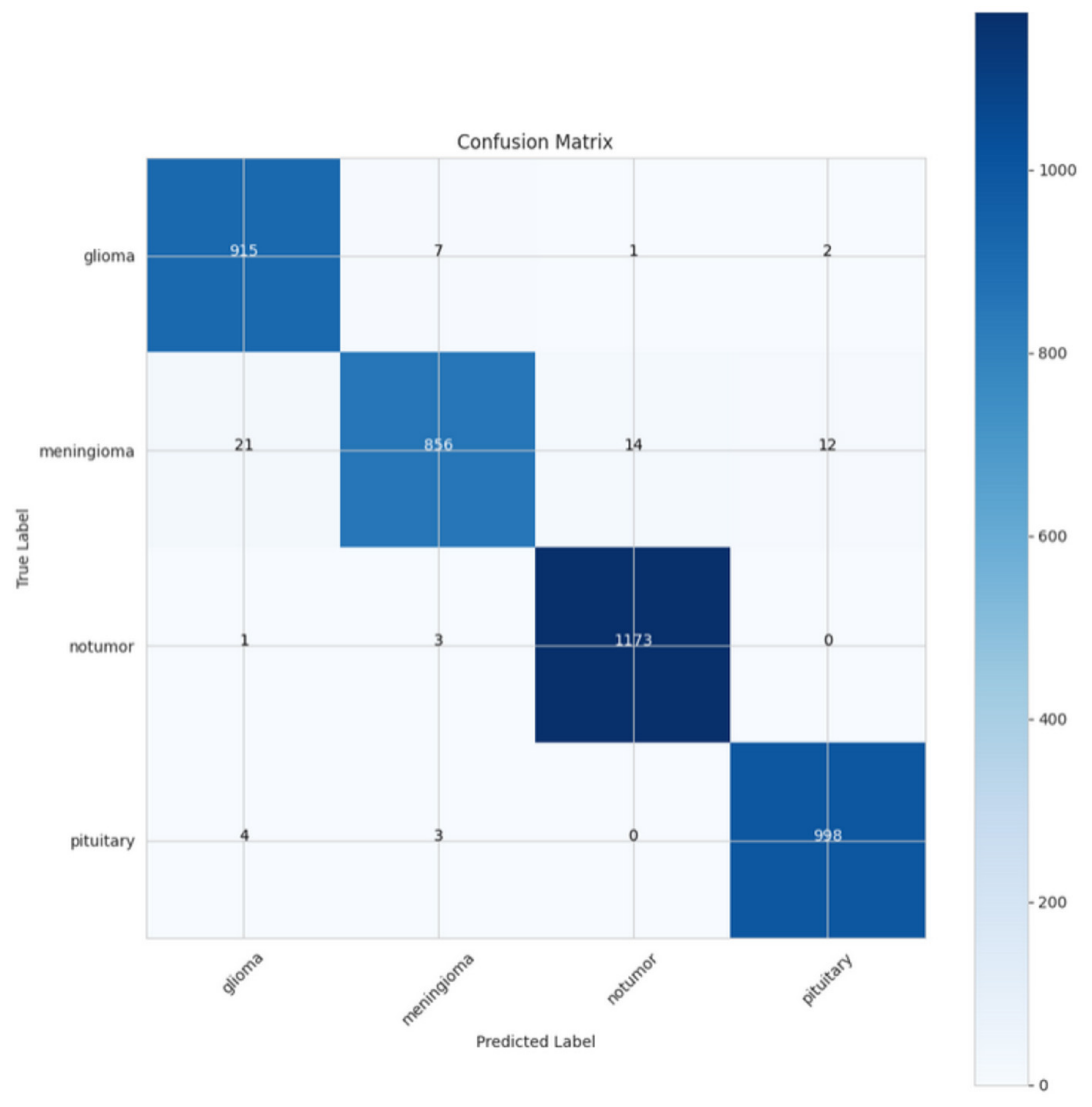
Confusion matrix of CerevianNet for Dataset-5.

The confusion matrix provided in Figure 20 shows how well the model performed on four classes: glioma, meningioma, non-tumor, and pituitary tumors. For glioma, the model correctly predicted 915 instances, with only seven misclassified as meningioma, one as non-tumor, and wo as pituitary. For meningioma, there were 856 total correct predictions with 21 misclassified as glioma, 14 as non-tumor, and 12 as pituitary. In the non-tumor category, the model predicted correctly in 1,173 cases with only one misclassified as glioma and three as meningioma. Finally, for the only 998 instances predicted correctly in pituitary, with four misclassified as glioma, and three as meningioma and as no non-tumor. Overall, the model performed phenomenally in each of the classes, and especially in the non-tumor and pituitary class, where there were nearly perfect predictions. Although the glioma and meningioma also have very high accuracy, there was some confusion between glioma and meningioma, and pituitary and meningioma. Regardless of this small misclassification, the model overall performed very well, identified the differences between the tumor types and the non-tumor.

**Figure 21:**
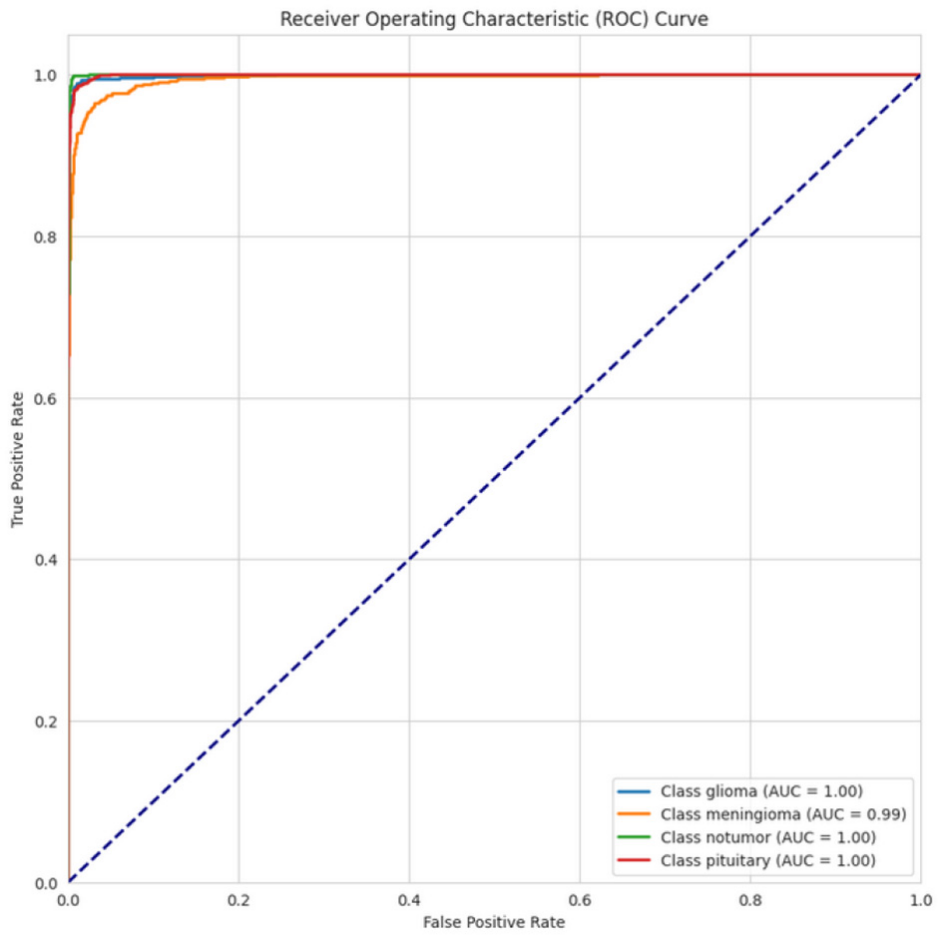
ROC-AUC curve of CerevianNet for Dataset-5.

Figure 21 displays the ROC curve for the performance of four classes: glioma, meningioma, non-tumor, and pituitary. All classes have high AUC, with glioma and pituitary achieving perfect AUC scores of 1.00, and very strong performance for meningioma (AUC = 0.99) and non-tumor (AUC = 1.00). The curves are all well above the diagonal baseline (perfect discrimination) to show the strong ability of the model to classify all the classes correctly. After running our model on different datasets, we observed that our model did not yield better validation and testing accuracy in a smaller dataset, but in a larger dataset, it yielded good testing and validation accuracy, and it also had low testing and validation loss.

The performance of CerevianNet differed greatly across the five datasets in Table 2. Dataset-1 (3,064 samples), which is only moderately small for machine learning data, achieved very high training accuracy (100%) and shows very strong generalization, achieving 98.04% validation accuracy and 96.74% testing accuracy, along with relatively low loss values (68.08 for training with zeros across the board for both validation and testing), confirming that the model is stable. Dataset-2 (7,023 samples) also achieved very positive results, maintaining 99.92% training accuracy and testing accuracy (97.1%). which is slightly higher than Dataset-1, confirming that the model can generalize well on larger datasets. The generalization for Dataset-3 (3,264 samples) and Dataset-4 (a combination of 5,859 samples) is very poor; both are suffering from high overfitting, achieving training accuracy that is near 100%, but dropping off significantly to validation and testing accuracies of approximately 74–76%, and with the respective very high validation and testing losses, which reflects that these models weakly generalize. Finally, Dataset-5 (40,000 samples), which is the largest dataset, achieved the most balanced and reliable results, achieving 99.67% training accuracy and with positive validation (98.17%) and testing accuracy (98.3%), and near-zero loss values (Calibrated = True; 3.1040, 5.8988, and 7.1897 losses respectively).

**Table 2:** Analyzing the performance metrics of CerevianNet across different datasets.

**Dataset number**	**Number of data**	**Training accuracy**	**Training loss**	**Validation accuracy**	**Validation loss**	**Testing accuracy**	**Testing loss**
Dataset-1	3,064	100%	0.197	98.04%	0.244	96.74%	0.274
Dataset-2	7,023	99.92%	0.117	98.16%	0.186	97.1%	0.22
Dataset-3	3,264	99.99%	0.214	74.11%	2.422	75.63%	2.61
Dataset-4.1 + 4.2	5,859	99.34%	0.178	74.05%	1.039	76.58%	1.08
Dataset-5	40,000	99.67%	0.057	98.17%	0.102	98.30%	0.111

In the fourth testing of our proposed model, we used two different datasets (Dataset-4 after merging). However, we had omitted some of the images from both datasets. The fact that this is a manually created dataset, and that we wanted to balance the number of samples across classes, meant we had a greater number of samples for some classes than we did for others, which is data balance. In such a situation, our proposed model, or any decent model, will inevitably face challenges. Secondly, for the third testing, we used dataset-3, which had fewer samples compared to the other four datasets. The small sample size also impacted performance; on the other hand, dataset-5 had the most samples, showed the greatest results, and provided the best image datasets. Our proposed model is very efficient; therefore, it relies heavily on a large amount of data for training. Dataset 5, by far, has the advantages in terms of data size. In summation, CerevianNet performs very well when trained on sufficiently large datasets; whereas when trained on smaller datasets or when the data are imbalanced, CerevianNet trained suffers from overfitting and instability.

### 4.1 Difference models performance on same dataset

**Figure 22:**
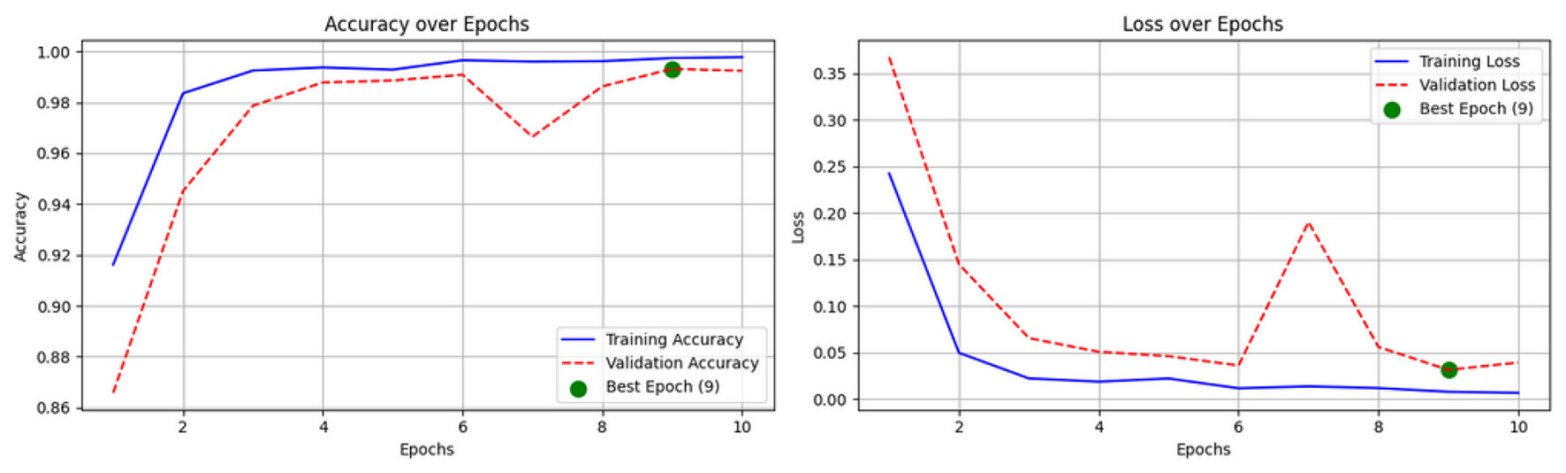
Training and validation accuracy and loss curve of Xecption for Dataset-1.

Since Dataset-1 is our main dataset for testing and evaluation purpose of our model we tested some light weight models on this dataset and how those models performs so firstly we select Xecption and train this model for 10 epochs with the same hyperparameters and optimization and in Figure 22 we achieved best validation accuracy of 98.9% and training accuracy 99.97% in 9 epochs with Validation loss 0.03 and training loss 0.015 but it training time higher than our CerevianNet.

**Figure 23:**
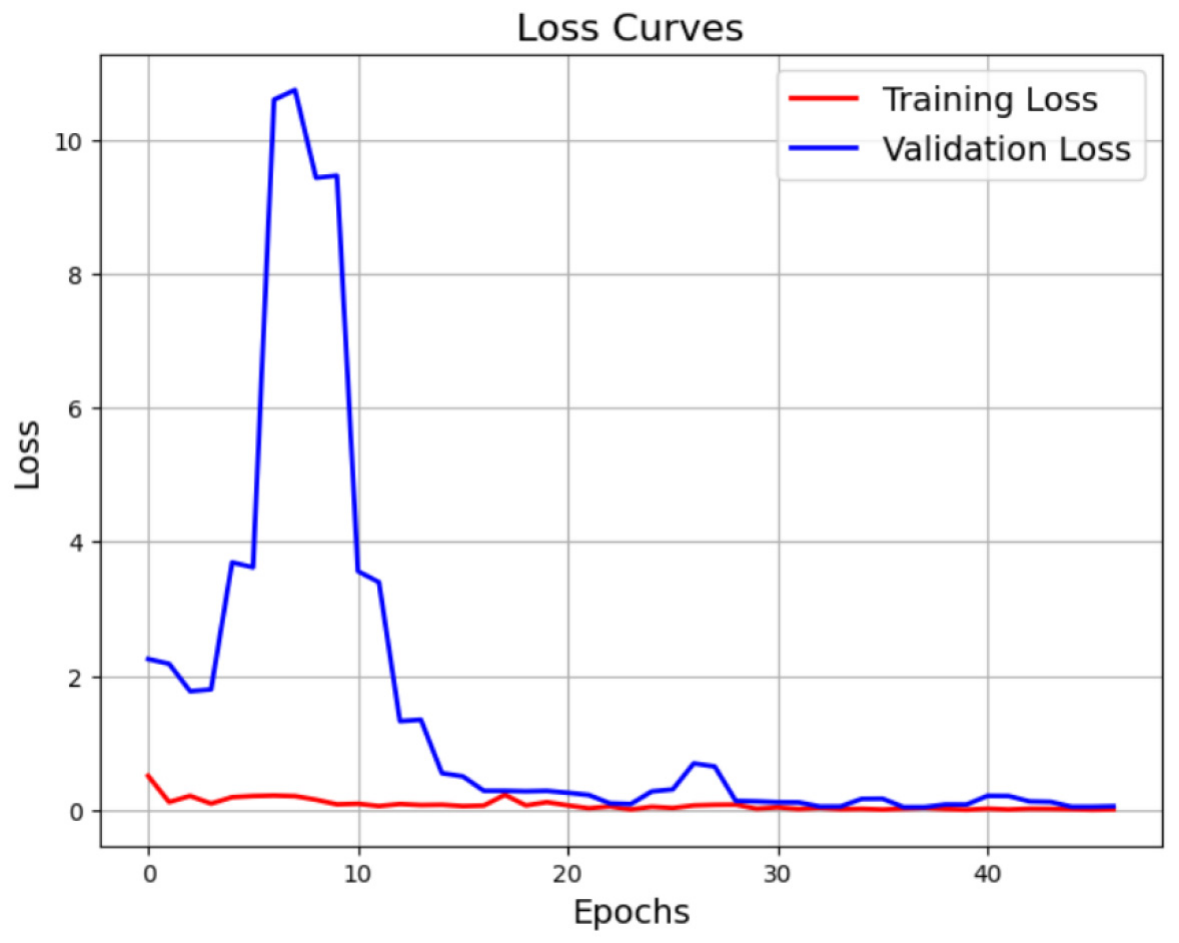
Training and validation loss curve of ResNet50 for Dataset-1.

The confusion matrix shown in Figure 23 is the performance of a classification model aiming to predict the type of brain tumor (glioma, meningioma, non-tumor, and pituitary). For the confusion matrix, the rows are the true labels, and the columns are the predicted labels. The majority of the predictions are correct, hence the strong performance by the model. The glioma class shows that the model predicted glioma with 298 instances correctly, misclassifying very few into the meningioma (1) or pituitary (1) classes. The meningioma class shows that the model predicted meningioma with 300 correct, misclassifying meningioma instances to the glioma (2), non-tumor (1), or pituitary (3) classes. The non-tumor class was predicted with perfect classification (405 instances) with no prior classification instances as false positives or negatives. The pituitary class had 299 instances recognized as predictions, with only one misclassified into the meningioma class. Overall, the model performs extremely well, as evidenced by the few misclassifications overall between glioma and meningioma; overall accuracy is high; and the model performs flawlessly at correctly identifying non-tumor and pituitary cases.

**Figure 24:**
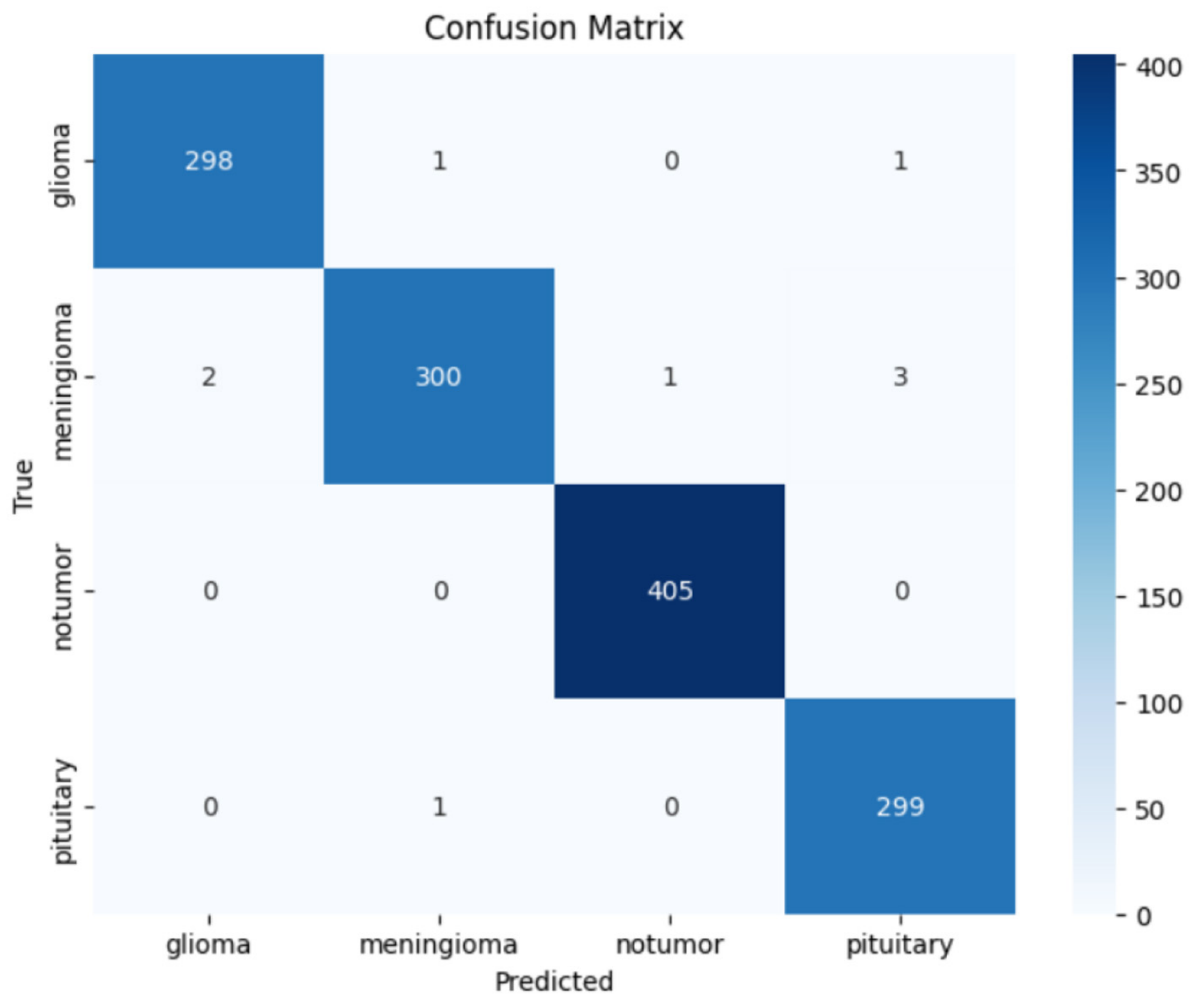
Confusion matrix of Xecption for Dataset-1.

**Figure 25:**
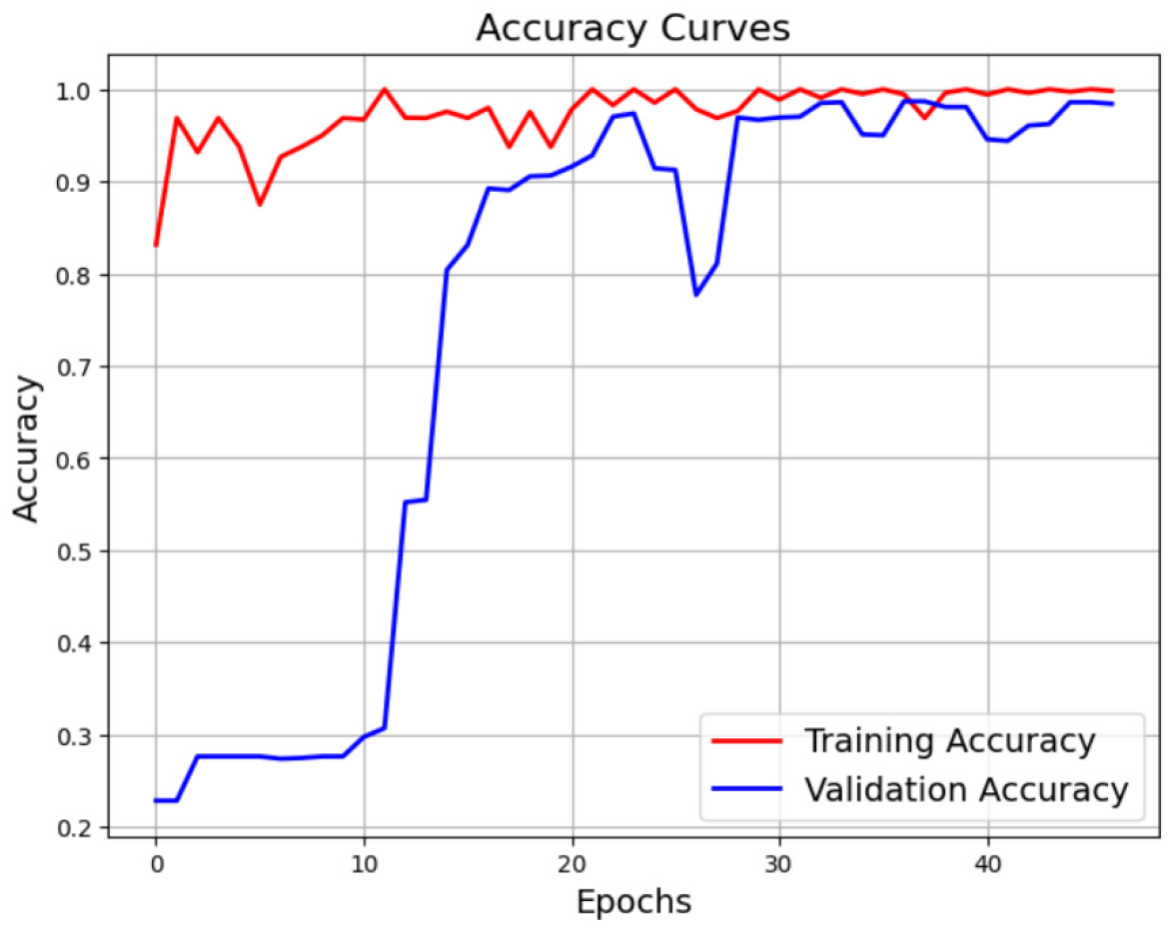
Training and validation accuracy curve of ResNet50 for Dataset-1.

After that, we used ResNet50 on the same dataset for 45 epochs with same optimization and hyperparameters; in Figure 24, it achieved training accuracy 99.99% and validation accuracy 99.1%. In Figure 25, it achieved validation loss 0.01 and training loss 0.001. As we know, ResNet50 is a lightweight model that makes it faster than Xception in training time but slower than our CerevianNet.

**Figure 26:**
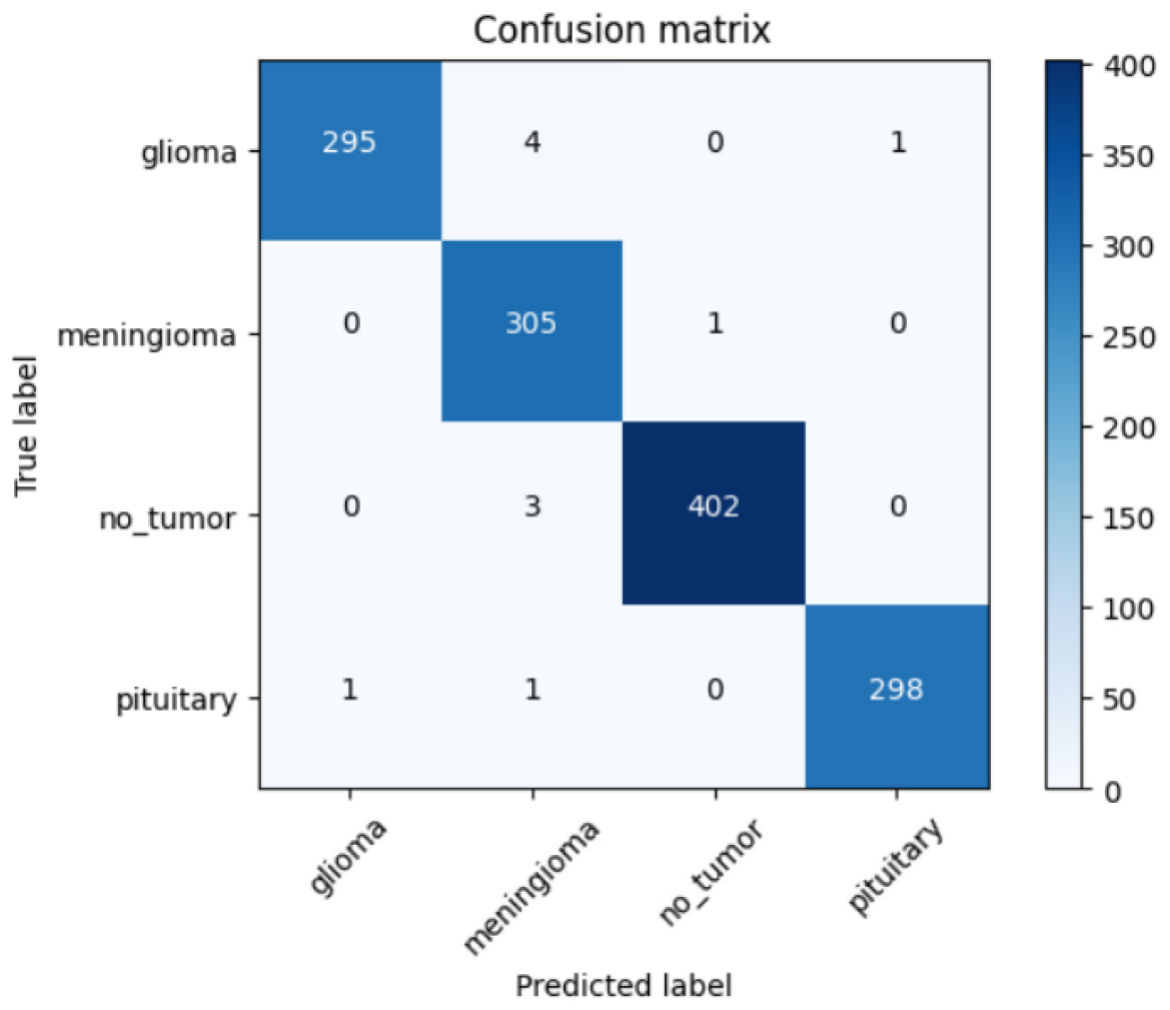
Confusion matrix of ResNet50 for Dataset-1.

The confusion matrix, shown in Figure 26, summarizes the performance of one of the classification models used to predict brain tumor types such as glioma, meningioma, no_tumor, and pituitary. For glioma, the model predicted the correct class 295 times. There were four predictions where glioma was misclassified as meningioma, one misclassified as pituitary and none as no_tumor. For meningioma, the model predicted the correct class 305 times and misclassified one no_tumor and one pituitary. The model predicted no_tumor correctly 402 times, misclassifying three as meningioma and none as glioma or pituitary. Finally, for pituitary, the model predicted the correct class 298 times, misclassifying one as glioma and one as meningioma. Overall, the model classified correctly most of the time, and had a very vary of mis-classifications particularly between glioma and pituitary, with the no_tumor class predicted almost perfectly.

**Figure 27:**
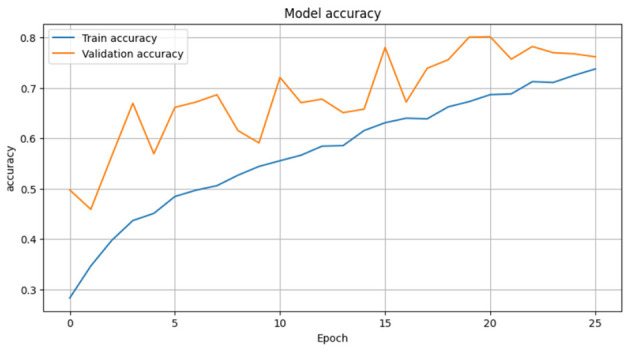
Training and validation accuracy and loss curve of EfficientNetV2 for Dataset-1.

**Figure 28:**
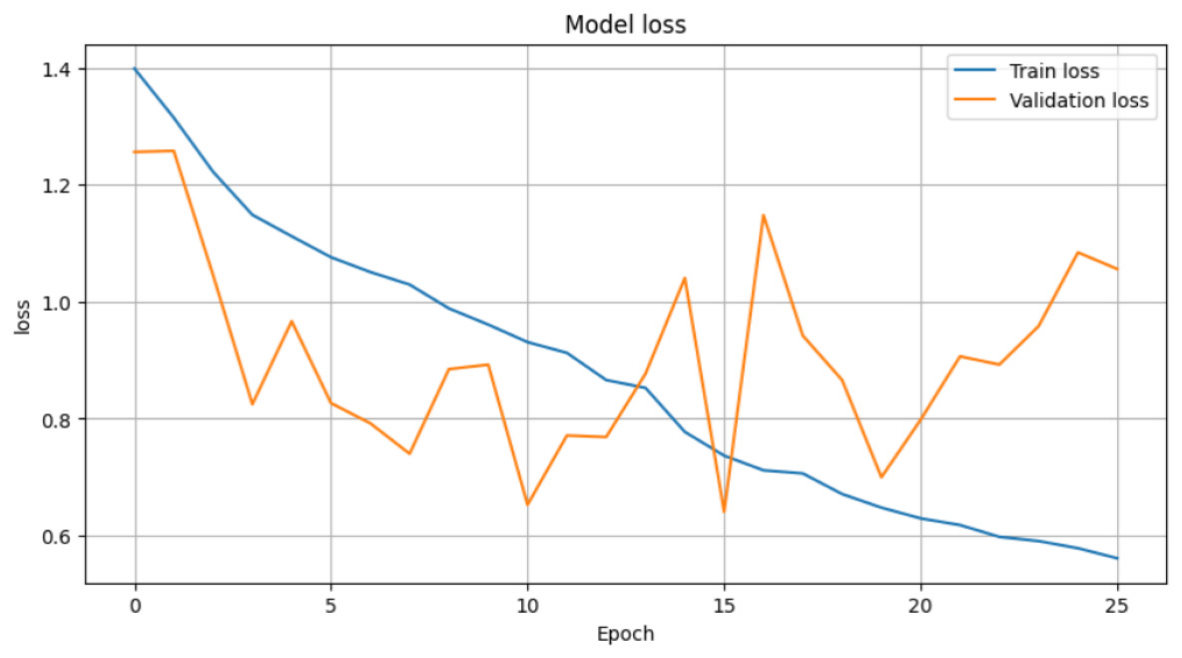
Training and validation accuracy and loss curve of EfficientNetV2 for Dataset-1.

We also applied EfficientNetV2 on that same dataset, which we trained for 25 epochs with the same optimization and hyperparameters. As depicted in Figure 27, it achieved the highest 80% validation accuracy with 69% training accuracy in 19 epochs. At that time, in Figure 28, the validation loss was 0.7, and the training loss was 0.65. it also faster than Xecption but less than our CerevianNet.

**Figure 29:**
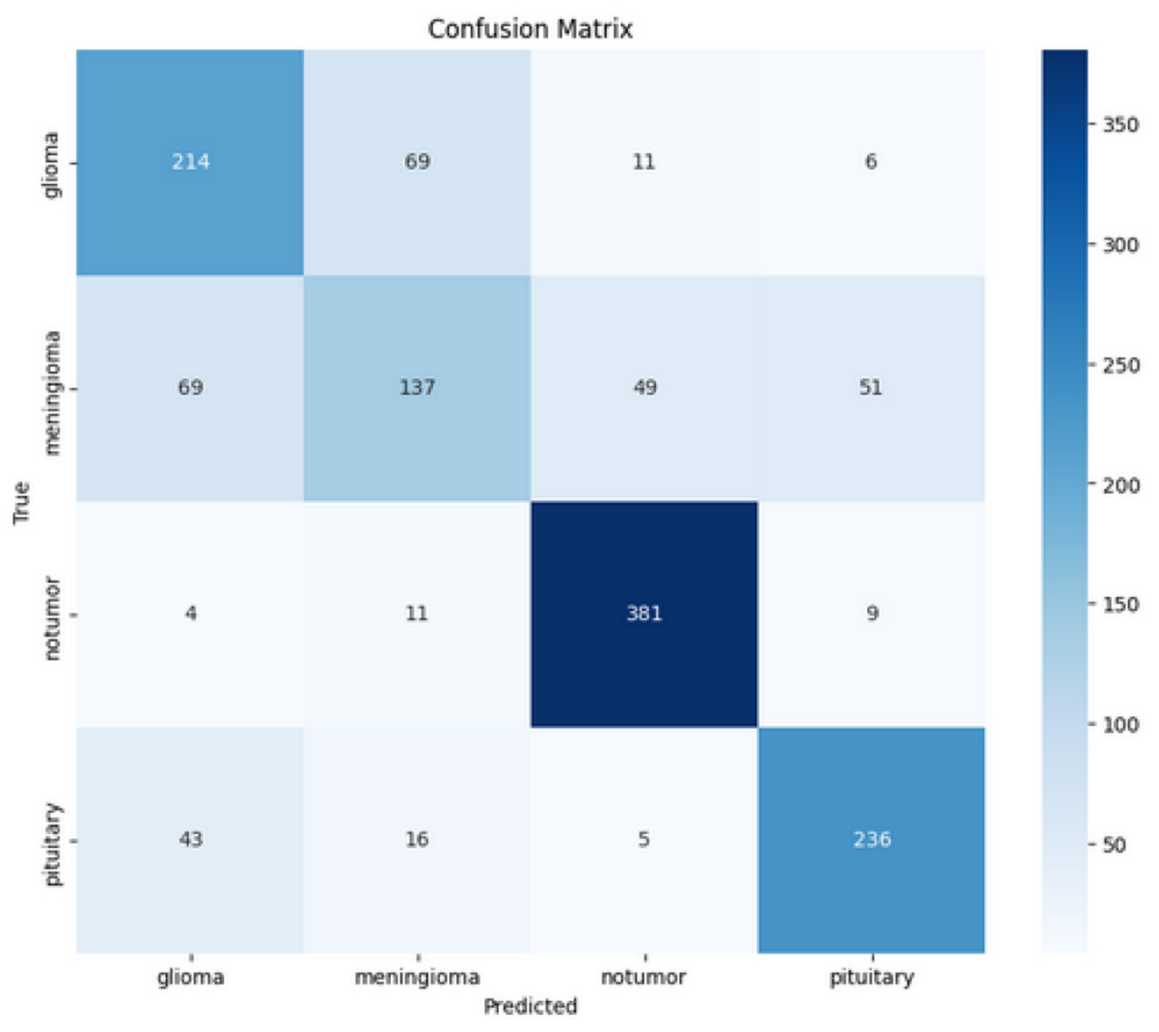
Confusion matrix of EfficientNetV2 for Dataset-1.

The confusion matrix shown in Figure 29 contains the classification results of a model used to predict four classes of cases: glioma, meningioma, non-tumor, and pituitary. Each row of the confusion matrix represents the true class, and each column of the confusion matrix represents the predicted class. The model is reasonably successful with the class “non-tumor,” as it made 381 correct predictions, though some of those classified as non-tumor were glioma (4), meningioma (11), and pituitary (9). The model is less successful with glioma and meningioma; it correctly predicts glioma (214 times) but misclassifies cases as meningioma (69), non-tumor (11), and pituitary (6). Within the meningioma class, the model correctly predicts results (137), misclassifies glioma (69), non-tumor (49), and pituitary (51). The model correctly predicts the class pituitary (236), but misclassifies as glioma (43), meningioma (16), and non-tumor (9). These misclassifications show the model where it is predicted classes lack performance, and for glioma, meningioma, and pituitary, which will be vital for a model.

**Figure 30:**
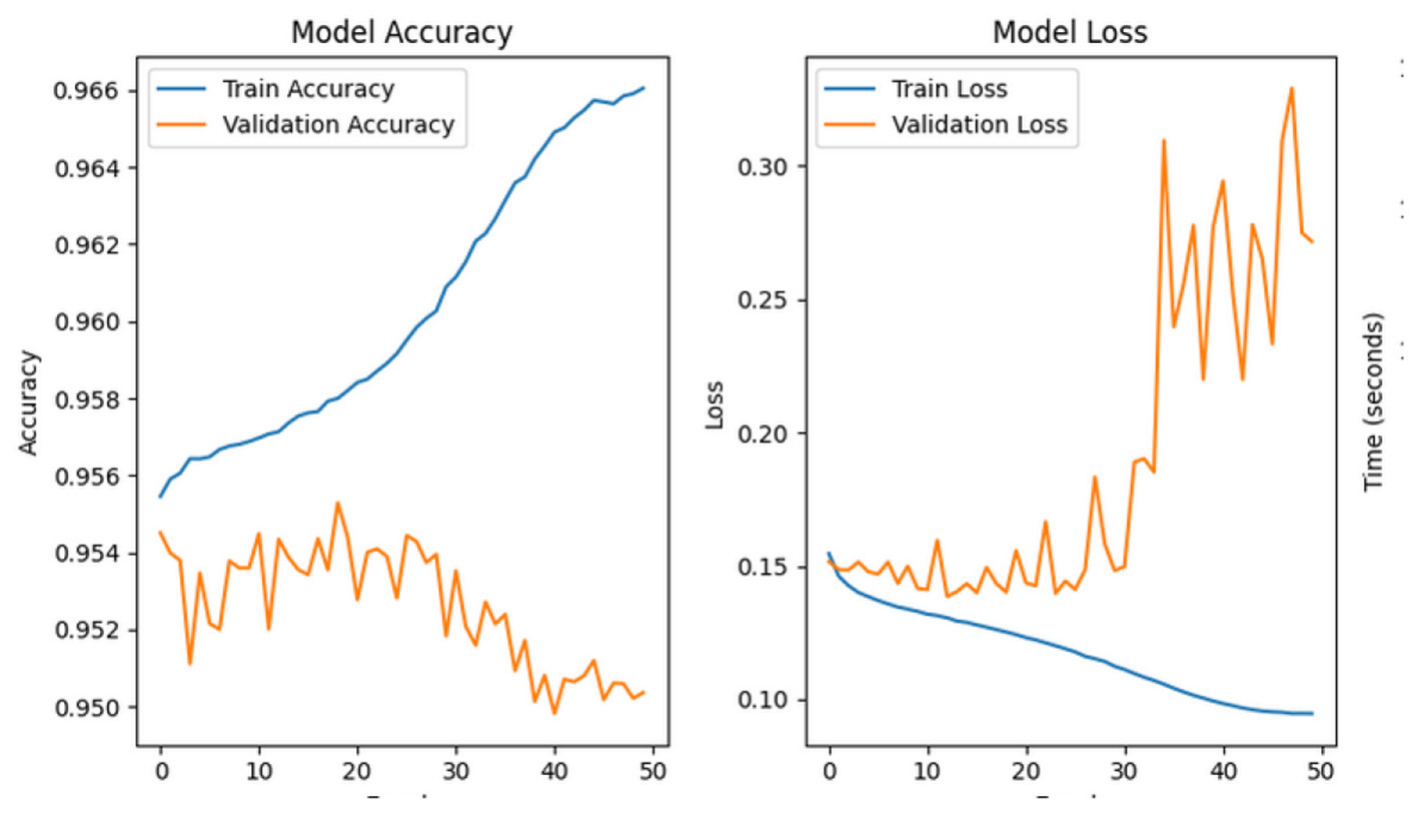
Training and validation accuracy and loss curve of SquuezeNet for Dataset-1.

We also trained SqueezeNet on that same dataset, and we trained for 25 epochs at the same optimization and setup, where, as depicted in Figure 30, it achieved 96.66% on training accuracy and 95.1% on validation accuracy. During that time, it achieved 0.1 on training loss and 0.27 on validation loss.

The ROC curve depicted in Figure 29 shows the performance of four classes: glioma, meningioma, non-tumor, and pituitary. All classes have high AUC values, with glioma and pituitary achieving perfect AUC scores of 1.00, while meningioma (AUC = 0.99) and non-tumor (AUC = 1.00) also perform excellently. The curves lie well above the diagonal baseline, demonstrating the model's strong ability to accurately classify all classes.

Table 3 compares deep learning models for brain tumor detection. It focused on parameter efficiency, test performance, and computational trade-offs. The Swin Transformer has 87.77 M parameters. It needs nearly 30 times more computing resources than the proposed CerevianNet. ResNet101 achieves a competitive accuracy of 99.22%. However, it has a high operational cost with 44.5 M parameters. Similarly, the Xception model has 22.9 M parameters. EfficientNetB3 has 12.23 million. They achieve near-perfect accuracies of 99.61 and 99.11%, respectively. They have 7.7 and 4.1 times more parameters than CerevianNet, respectively. This shows that small accuracy improvements (1–1.6%) come at very high computational costs.

**Table 3:** Comparative performance metrics of deep learning models.

**Models**	**Params**	**Test loss**	**Test accuracy**
Swin transformer	87.77 M	0.3875	0.84
AlexNet	62.3 M	0.1358	0.96
Resnet101	44.5 M	0.1044	0.99
Resnet50	25.6 M	0.3419	0.98
Xception	22.9 M	0.0809	0.99
EfficientNetB3	12.23 M	0.0922	0.99
DenseNet121	7.97 M	0.7230	0.97
**CerevianNet (Our proposed)**	**2.98M**	**0.0508**	**0.98**

CerevianNet's lightweight design achieves excellent computational efficiency while keeping good diagnostic performance. This makes it uniquely suitable for real-world medical use. Models like Xception and EfficientNetB3 performed slightly better in accuracy. However, their large sizes are much bigger than CerevianNet. This makes them impractical for hospitals or clinics with limited computing resources. CerevianNet's efficiency is very different from high-accuracy models like Xception and ResNet101. These models need 7.7 to 15 times more computing power for only small performance gains. This balance makes CerevianNet a practical choice for hospitals. Fast and reliable results are more important than slightly better accuracy there. By greatly reducing computing needs without losing reliability, CerevianNet combines accuracy with real-world usability in medical AI.

### 4.2 Performance comparison: our proposed model and existing methods

Table 4 presents a comparative analysis of our proposed model, CerevianNet, with existing works in terms of classification accuracy. The models evaluated include AlexNet, ARM-Net, Parallel CNN, Custom CNN, and FTVT-b16. Among these, our proposed model achieves the highest accuracy of 98.09%, surpassing all others. Notably, AlexNet performs the lowest with 96.2%, and FTVT-b16 achieves a close 98.07%. On the other hand, transformer-based models like ViT achieve 97% accuracy while PBViT, FT-ViT, and RanMerFormer achieve accordingly 95.8, 98.13, and 98.86%. Among them, EFFResNet achieves the highest accuracy with 99.31%. Despite similar accuracies, the competing models involve more complex or ensemble-based architectures, while CerevianNet offers superior efficiency and simplicity. This advantage made it more practical for deployment. This demonstrates the effectiveness and efficiency of our approach in improving classification accuracy.

**Table 4:** Comparison of our proposed model with existing work.

**Author**	**Model**	**Accuracy**
Kumar et al. (33)	AlexNet	96.2%
Dutta et al. (34)	ARM-Net	96.64%
Rahman and Islam (35)	Parallel CNN	97.60%
Khan and Park (36)	Custom CNN	97.52%
Reddy et al. (37)	FTVT-b16	98.07%
Sundar et al. (38)	ViT	97%
Asiri et al. (39)	FT-ViT	98.13%
Chauhan et al. (40)	PBViT	95.8%
Wang et al. (41)	RanMerFormer	98.86%
Hussain et al. (42)	EFFResNet-ViT	99.31%
**Our proposed**	**CerevianNet**	**98.09%**

## 5 Future work

This study highlights the potential of our proposed model for enhancing brain tumor classification using MRI images. Future research could focus on improving its robustness and accuracy by integrating multimodal data, such as genetic information, histopathological findings, and patient demographics. Incorporating explainability tools like Grad-CAM can improve transparency by visualizing critical areas influencing the model's decisions. This will be essential for clinical adoption and trust. Periodic retraining with updated MRI datasets, including rare or emerging tumor types, can ensure the model adapts to advancements in imaging technology and changing clinical practices. Collaborating with global healthcare institutions to validate the model across diverse populations and MRI systems can further strengthen its reliability.

## 6 Conclusion

In this study, we proposed and validated a lightweight custom CNN model, CerevianNet, for multi-class brain tumor classification using MRI images. The model demonstrated high classification accuracy with a minimal number of parameters. This ensured computational efficiency and suitability for resource-constrained devices. Our methodology included robust data preprocessing, the development of a custom CNN architecture, and evaluation through diverse metrics. This highlighted its capability to classify different tumor types effectively. Future enhancements could involve leveraging larger and more diverse datasets to further improve model robustness and generalization, as well as exploring fine-tuning techniques and multimodal data integration. Collaborating with medical professionals for clinical validation will ensure the model's reliability in real-world scenarios. This will contribute to more accessible, precise, and efficient diagnostic support in healthcare.

The newness of CerevianNet is not just in how parameter efficient it is but also in its architecture's design choices made specifically to enhance its brain tumor classification ability. The model utilizes a lightweight architecture, growing its filters size progressively, (16, 32, 64, 128 filters) in its convolutional blocks; this allows the model to learn low and complex features of the MRI images of tumors simultaneously, preserving crucial details. Moreover, all the convolution layers utilize Batch Normalization, and all activation functions make use of LeakyReLU; these characteristics help with faster convergence and minimize the chance of gradient vanishing. Moreover, the model utilizes Dropout layers, along with L2 regularization, which contribute to the avoidance of overfitting, improving the model's ability to generalize better to unseen data, primarily when deployed in narrow periodontal areas, such as health care. Therefore, these design choices and thoughtful character selections help CerevianNet to maintain rigidity in learning tumor features and achieve high classification rates, even with comparatively low parameters in its specifications.

CerevianNet has experienced failure cases in smaller datasets, particularly with glioma tumors, which were sometimes misclassified as pituitary tumors or no tumor at all. Gliomas and pituitary tumors can be similar in some cases, and when a model is applied to smaller datasets that do not contain sufficient unique examples, it may not capture the subtle differences between the two types. Misclassification of gliomas as no tumor can be more pronounced with low-grade gliomas, as, in the earlier phases, low-grade gliomas may not exhibit anything that the trained model deems as distinctive. The situation was compounded by the situation of smaller datasets, both because of less diversity in the training data as well as the smaller size of the dataset. The availability of increased datasets through data augmentation or synthetic data generation could reduce the misclassification issue since the model would have access to more unique examples. This would allow the model to better differentiate between similar categories of tumors, as well as help the model detect subtle gliomas correctly.

In terms of clinical use, CerevianNet has high potential for real-time, inexpensive tumor classification. The compact nature of its architecture allows it to be used in telemedicine contexts, which can be deployed in remote healthcare contexts and assist medical practitioners in assessing brain tumors rapidly, correctly, and financially feasibly—even within the constraints of limited resources. CerevianNet can be placed into established clinical workflows as a prescreening step that allows radiologists to focus on more complicated assessments. In terms of edge deployment, CerevianNet was engineered to be performant on low-power/mobile devices and tablets, but also MRI machines with limited memory and computing ability. Because its number of parameters is reduced (6.5M), the model consumes fewer resources, likely found on mobile devices (phone/tablet/computers), enabling it to run efficiently without overburdening the device. Although many considerations apply to edge deployment, such as quantization steps, can reduce memory and computation burden, and provide a boost in inference speed, as well as reducing overhead in energy consumption. Thus, CerevianNet is capable for edge devices, where it works well on mobile CPUs or embedded systems in a health care setting.

## Data Availability

The original contributions presented in the study are included in the article/supplementary material, further inquiries can be directed to the corresponding author.
